# AI-Driven intrusion detection and prevention systems to safeguard 6G networks from cyber threats

**DOI:** 10.1038/s41598-025-21648-5

**Published:** 2025-10-29

**Authors:** P. Chinnasamy, Sarojini Yarramsetti, Ramesh Kumar Ayyasamy, Ella Rajesh, Vijayasaro V., Digvijay Pandey, Binay Kumar Pandey, Mesfin Esayas Lelish

**Affiliations:** 1 Department of Computer Science and Engineering, School of Computing, Kalasalingam Academy of Research and Education, Srivilliputtur, Tamil Nadu India; 2 Department of Computer Science and Business Systems , Nehru Institute of Engineering and Technology, Coimbatore, 641 105 India; 3 Faculty of Information and Communication Technology , Universiti Tunku Abdul Rahman , Kampar, Perak, Malaysia; 4 Department of Chemistry, Aditya University, Surampalem, Andhra Pradesh India; 5 Department of ECE, Guru Nanak Institute of Technology, Hyderabad, India; 6 Department of Technical Education Uttar Pradesh, Government of U.P., Lucknow, India; 7 Department of Information Technology, College of Technology, Govind Ballabh Pant University of Agriculture and Technology Pantnagar Udham Singh Nagar, Uttarakhand, India; 8 Department of Statistics College of Natural and Computational Science , Mizan-Tepi University , Tepi, Ethiopia

**Keywords:** 6G network, Cyber-attack monitoring, Intrusion detection system, Machine learning algorithm, Binary wolf optimization, Computational models, Data processing, Hardware and infrastructure, Image processing, Machine learning, Programming language

## Abstract

Sixth-generation (6G) wireless networks, which boast previously unheard-of capacity, reliability, and efficiency, are projected to begin testing and implementation as early as 2030. To meet the demands of new applications, the emphasis is currently on developing 6G networks. The advent of 6G presents additional difficulties, especially in intrusion detection, where sophisticated attacks call for cutting-edge security measures. This research proposes a novel technique using a machine learning algorithm in a 6G network cyber-attack monitoring and intrusion detection system. Here, the 6G network has been monitored, and intrusion detection for cyberattack using blockchain federated Gaussian multi-agent Q-encoder neural networks (BFGMAQENN). Then, the 6G network has been optimized using whale swarm binary wolf optimization (WSBWO). The experimental analysis has been carried out for various cyberattack datasets regarding detection accuracy, data integrity, scalability, communication overhead, and network efficiency. The proposed model attained detection accuracy of 97%, data integrity of 94%, scalability of 93%, communication overhead of 60%, and network efficiency of 98%.

## Introduction

 Infrastructure implementation for the fifth generation of mobile networks has commenced, and its widespread adoption is anticipated in the next few years. Current works by practitioners and academicians are geared towards 6G infrastructure to meet the acceptable application levels in the next ten years. Several case studies show that 5G networks present limitations in data rate, latency, global reach, and several other parameters. Advanced applications like immersive reality, holographic telepresence, and digital twins will make the full benefits of 6G network systems possible. High data transfer speeds, energy saving, low delays, and connection of various devices are the primary advantages of 6G networks over 5G networks. It is difficult to accomplish zero-day attack detection because suspicious activity is discovered daily^1^. These complex breaches can cause significant harm, increasing the difficulty of defending against them with current intrusion detection systems (IDSs). Cyberattacks have increased in magnitude and scope in recent years, causing significant losses and negative consequences. By 2021, cyberattacks might cost up to $3 trillion, according to forecasts made by cyber security experts. Furthermore, the volume of data that data-driven companies store on both public and private clouds by 2022. Additionally, IoT attacks can potentially cause significant damage^2^. Computer system defects, security measures with lax security requirements, and a lack of awareness of breaches and crimes have all contributed to an increase in several targets for network attacks. IDS was implemented to examine data throughout the network and notify users of potentially harmful intrusion activities. IDSs send out notifications when they identify known threats or unexpected activity and search for signs of potentially harmful behaviour, network packets that allow unauthorised users to access the system, and cyber defences against disruptive activity. An intrusion detection system in 6G networks seeks to identify anomalous network traffic patterns, take prompt action to prevent security breaches, and proactively monitor network traffic^3^. Malicious software backdoors and hacking remained the most common security issues. In addition, ransomware increased by around 13%, the same amount over the preceding five years. Network security tool implementation is essential in healthcare to safeguard patient information from illegal access and disclosure by legal, ethical, and medical requirements. Because cyberattacks are becoming more frequent and complex every year and are unpredictable, it is essential to design an efficient network security solution to safeguard data and identify such threats. A Network Intrusion Detection System (NIDS) security technology examines network data flows to identify dangers and stop malicious requests^4^. NIDS can be divided into two kinds according to the detection method: signature-based and anomaly-based. Signature-based NIDS uses a database of existing patterns or signatures to pinpoint possible threats.

This type has difficulties identifying novel or zero-day attacks but can detect low computational risk. At the same time, anomaly detection NIDS has the advantage of detecting new threats or unusual behaviour but has a high false positive rate. NIDS must be developed to detect novel and known attacks efficiently and accurately. In the recent past, anomaly-based intrusion detection systems have attracted huge consideration. The probability of discovering new attacks is high due to the existing variety of attacks. Ongoing development and designs of multilayer learning algorithms for detecting anomalies and intrusions. the development and implementation of various device learning systems for over-intrusion detection^5^. The algorithms used in these techniques focus on conducting fact-based analyses that do not rely on specific functionalities. This approach is particularly beneficial when the site’s users or traffic patterns are diverse. Most of the time, the principal cause is the failure to implement anomaly-based intrusion detection systems. With the heavy data burden level, the demand for the connections is high, problems become localised, and scale increases quickly. In tandem with the risk factors, artificial intelligence technologies in the 6G network are under attack as a result of the expeditious evolution of the system. 6G networks, concerning learning on different edge devices, are not immune from data poisoning and other distributed AI risks. Nevertheless, deep probabilistic ML has recently shown potential in tackling such attacks through uncertainty estimates. However, data poisoning remains a threat as a hostile actor can plant a few intact, noise-free, highly precision, optimally augmented samples into the training dataset purposed to avoid degradation. The proliferation of such threats is understandable, given that the number of IoT devices is expected to surge within the 6G era, which correlates with rapid advancements in Wireless Sensor Networks. Broadly, IoT embodies fluid ecosystems that are continuously churned and generate diverse data types that undergo complex and outlier variations over time. Therefore, we expect that future security measures should be able to quickly adjust to changes over time and detect undiscovered malware strains. However, traditional machine-learning approaches are isolated^6^.

### Major contributions of the work

- To suggest a unique machine learning algorithm-based intrusion detection and cyberattack monitoring method for 6G networks.
- The blockchain federated Gaussian multi-agent Q-encoder neural networks (BFGMAQENN) have been used to monitor the 6G network and detect intrusions for cyberattacks. Then, swarm binary wolf optimisation (WSBWO) optimised the 6G network. In a 6G setting, we suggest a software security architecture for large IoT networks that combines several security tiers and adaptive protection techniques.

## Related work

In recent years, many studies have focused on constructing intrusion detection systems. This collection of studies aims to pinpoint a few limitations and challenges experienced, including forecasting errors and volume of data problems. Kumar et al.^7^ claim that harmful activities such as Distributed Denial of Service (DDoS), Man in The Middle (MITM), spoofing attacks have increased the explosion of big data. A data dimensionality reduction strategy was proposed to lower the data dimension to increase the detection rate. Support Vector Machine (SVM) and Neural Network (NNet) were used as single classifiers, while Extreme Gradient Boosting (XGBoost) and Conditional Inference Trees (CTree) were used as ensemble methods. In the study by Ferrag et al.^8^, the ability of a Deep Neural Network (DNN) to detect abnormal behaviour in the Internet of Things was tested. Various validation techniques, including cross-validation and repeated cross-validation, were applied to different datasets, such as Coburg Intrusion Detection Data Sets-001 (CIDDS-001), University of New South Wales Network Based (UNSW-NB15) Network Intrusion Dataset, General Packet Radio Service (GPRS), to achieve a robust performance evaluation^9^. The search was carried out for each dataset to identify the most suitable Deep Neural Network (DNN) hyper-parameters. When Class was chosen as the outcome variable, the method achieved almost 100% accuracy on the CIDDS-001 dataset. Sedjelmaci et al.^12^ assessed the Long Short-Term Memory (LSTM) model’s performance using flow-based data from the CIDDS-001 dataset. Their results were then compared to those obtained from other standard classifiers. Kohli et al.^10^ analysed the skewed CIDDS-001 dataset from a machine-learning perspective, finding that both the k-nearest neighbour (k-NN) Classifier and k-means Clustering techniques performed well andplan to compare the CIDDS-001 dataset with other current benchmarking datasets to evaluate its complexity in future work. Muniyandi et al.^11^ applied a Long Short-Term Memory (LSTM) method to evaluate the CIDDS-001 dataset, which consists of External Server data. For this study, 33% of the data was reserved for the testing phase, while 67% was utilised for training. In contrast to the SVM, NB, and MLP approaches, the performance of LSTM outperformed the rest of the approaches. Most hyperparameter settings are presented, although no information is provided regarding the sequence length. Kong et al.^13^ introduced a novel weight-based ensemble learning algorithm (WBELA) to detect unauthorised communication within a vehicle’s Controller Area Network (CAN). Studies indicate that proposed approaches are more effective than existing techniques regarding precision, efficiency, and false alarm rate.

A possible limitation of this strategy is the reliance on simulated results for evaluation instead of live data. actual data. Alsubai et al.^14^ proposed a key for a signature-based wireless 6G IoT intrusion detection network. An intrusion detection system that relies on signatures provides wireless 6G IOT networks. This achieved an accuracy rate of 98.9% after applying three different techniques. One of the limitations of wireless 6 IoT networks is the implementation of signature-based intrusion detection systems, which cannot detect intrusions of unpredicted attacks, also known as zero-day attacks. Also, Hadi et al.^15^ developed a novel type of Anomaly Detection System of Communication Networks, 6G Networks (AD6GNs) based on Ensemble Learning (EL). It is, however, important to mention that overall accuracy of 99.5% (false alarm rate 0.0038) was achieved on the Network Security Laboratory-Knowledge Discovery in Databases (NSL_KDD) dataset, 99.9% (false alarm rate 0.0076) on the UNSW_NB15 dataset, 99.8% (false alarm rate 0.0009) on Canadian Institute for Cybersecurity Intrusion Detection System (CIC_IDS2017) dataset, and an overall accuracy of 99.95426% (false alarm rate 0.00113) was attained for Canadian Institute for Cybersecurity - Distributed Denial of Service 2019 (CICDDOS2019) dataset. Furthermore, the proposed approach has a potential flaw because it relies on historical records for its training. Hoang et al.^16^ developed a two-level security system. The first element consists of a reinforcement learning model designed to evaluate the trustworthiness of the messages. An advanced context-aware trust management strategy is also proposed to enhance the quality of these evaluations. In this second part, the overall effectiveness of the framework is enhanced due to the inclusion of the most appropriate evaluation technique^17^. All of his three subsystems are presented in this location. The first subsystem, titled the False Data Insertion (FDI) Subsystem, aims to Program adversaries such as attacks on self-driving cars. Second is Cyberattack Dataset Collection Subsystem (CDC). Data is generated and collected using Normal and Attack Mode simulation models^18^. Data Classification in Intrusion Detection Mechanism (IDM) Subsystem utilises various LSTM (long short-term memory) networks to detect various cyberattacks, including FDI, in vehicle control systems.

The model predicts if the sample data is normal or abnormal. Unlike other advanced models, the experimental results reported by Chinnasamy et al.^19^ showed that the model had high and efficient detection rates. Deep learning based on the one-dimensional Convolutional Neural Networks (CNN) architecture was developed by Bhuvaneshwari et al.^20^ to model NIDS for detecting four attack types in the CICIDS 2017 dataset. The four attack types include Port Scan, DoS Goldeneye, DDoS and DoS - Hulk. The accuracy when employing five CNN layers over 50 epochs was recorded as 98.96%. In their study, Son et al.^21^ performed feature engineering using a Convolutional Neural Network (CNN) and applied Deep Feature Synthesis (DFS). Features were generated through DFS, while principal component analysis (PCA) was used for dimensionality reduction. CNN, consisting of three to five convolutional layers, was utilised for binary and multiclass classification. Kumari et al.^22^ proposed a hybrid NIDS model incorporating CNN, LSTM, and their combination. The LSTM approach captured the temporal aspects of the characteristics, while the CNN technique focused on spatial elements. The method was evaluated using the CICIDS2017, UNSW-NB15, and Wireless Sensor Network DoS Detection Dataset (WSN-DS) datasets.

The CNN-LSTM-based model achieved the highest accuracy and detection rate among the approaches. Blika et al.^23^ presented a design for an intrusion detection system of Software Defined Networking employing a Gated Recurrent Unit Recurrent Neural Network (GRU-RNN). Preprocessing of dataset was done using Min-Max normalisation techniques. The L1-regularization method was employed to reduce overfitting. The overhead issues were quite high for the model to cope with. Abdelrahim et al.^24^ proposed a lightweight intrusion detection method which can work in real environments. Attributes were extracted using the wrapper attribute selection technique. SVM as well as Apriori association rule mining classify attacked and benign network traffic. Maiwada et al.^25^ classified and analysed decision algorithms for handovers based on factors like user equipment speed, cost implications, interference, and received signal strength. However, most of these decision algorithms do not consider the critical aspects. Gkonis et al.^26^ presented an effective semi-supervised model for increasing detection rates and decreasing false positive errors. Dimensionality of the data is reduced with use of PCA. New data is classified with five-fold cross-validation and k-nearest neighbour hyperparameter tuning.

The ACAE-RF model by Thanganadar et al.^27^ sought to exploit the strengths of both shallow and deep learning techniques. The ACAE (Asymmetric Convolutional Autoencoder) network is used to extract features. Random Forest classifier, used in ensemble learning techniques, is then used to segregate the network traffic data. Maiwada et al.^28^ investigate the challenges typical of 5G environments, such as high transaction volumes and diverse device requirements, discuss strengths and weaknesses of various scalability techniques in light of such an environment. Uysal et al.^17^ proposed the ‘UTTAMA’ method to improve detection precision for each attack category. The membership function helps minimise the dimensionality of the dataset. J48 is the classification algorithm used. A model HIDS developed by Hu et al.^29^ consists of three stages of data preprocessing – data reduction, attack category translation, data normalisation. Features were selected utilising the FSM model presented in FEI and MEI sections. OSVM is applied for outlier detection, while prioritised KNN is used to classify data. Tandra et al.^30^ assessed various machine-learning models using KDD99 dataset and found that the Random Forest (RF) algorithm outperformed others like SVMs, Naive Bayes, Logistic Regression, achieving an accuracy of 99.81%. Alshahrani et al.^31^ tested several methods in a separate study across four datasets, including CIDDS-001 and observed that RF performed best overall^32,33^, with an average accuracy of 94.94%, surpassing AdaBoost and Extreme Gradient Boosting techniques. In order to identify breaches in Internet of Things (IoT) settings, this research^34^ suggested a new method known as FLBC-IDS. It integrates Horizontal Federated Learning (HFL), Hyperledger Blockchain, and EfficientNet. By using HFL, the FLBC-IDS model is able to train models securely and privately across a large number of IoT devices, leading to decentralized data privacy and efficient use of resources^36^. developed an innovative Edge-based Framework that safeguards ITS against new dangers by combining Federated Learning (FL) with blockchain technology. Specifically, our suggested framework includes (1) an innovative distributed Edge-based design that enables numerous Edge nodes to safely cooperate while protecting their privacy, (2) a blockchain-based decentralized reputation system to ensure the FL process within the ITS is reliable and trustworthy. The authors^41^, proposed a new method for strengthening the safety of edge cloud computing networks that are enabled by IoT. It does this by analyzing data using designs such as Gaussian Bayesian transfer convolutional neural networks^23^ and blockchain-driven FL. Protecting user data and identity in consumer IoT apps is the goal of blockchain-powered FL. Throughput as high as 89%, latency as low as 71%, training accuracy as high as 91%, validation accuracy as high as 96%, and network security as high as 92% as compared with existing works. The existing model-based summary is shown in Table 1.

**Table 1:** Summary for existing model.

References/Authors	FS Methods and Number of Features	Classifiers Methods	Experimental Results	Cons
^11^	Employed RF EL in conjunction with correlation FS. For, this system chose 30, 35, and 40 FSs, respectively.	Using two modified classifiers, training them as AdaBoosting and bagging EL, and then combining them using the voting average method.	NSL_KDD has an accuracy of 99.6% with 0.004 FAR, UNSW_NB2015 has an accuracy of 99.1% with 0.008 FAR, and CIC_IDS2017 has an accuracy of 99.4% with 0.0012 FAR.	Due to combination of two EL strategies for separating and spreading legitimate or suspect network traffic attacks, the complexity time measurement took excessively long.
^30^	N/A	Rule learner-based EL and DT.	The results demonstrate that the IDS classifier techniques have FPR and highest classification accuracy for accuracy, DR, and FAR.	inaccurate findings and multiple attacks that went unnoticed. Additionally, a long period of time spent searching with lowest FNR and accuracy.
^31^	LDA	KNN is used in a two-tier anomaly-detection model.	83.24% accuracy, 4.83% FAR, 82% TPR, 5.43 FPR were evaluated experimentally.	required additional execution time. inadequate handling of anomaly dataset’s network imbalance.
^32^	Information gain uses ten features for multiclass and thirty-two features for binary classes.	Adaptive Greedy randomised hybrid RF.	With data gain achieving an accuracy of 78.9035%, the accuracy is 85.0559%.	Less accuracy and high FAR.
^33^	DT for FS.	ML methods for hybrid wireless sensor networks that focus on energy efficiency and anomaly detection.	According to the trial findings, the accuracy was 95%, precision was 94.00%, the recall was 98.00%, F1-Score was 96.00%.	A high FAR and a long search time.
^34^	A genetic algorithm serves as foundation for wrapper method’s feature selection.	For classification, many classifiers are employed.	With 98.75%, 96.64%, and 98.93% DRs, the findings demonstrated accuracy of 98.99% for CIC_IDS17, 98.73% for NSL_KDD, and 97.997% for UNSW_NB15.	inaccurate findings and multiple attacks that went unnoticed.
^33^	To choose best subset features, RF was utilized. NSL_KDD, CIDDS-001, and CIC_IDS2017 were utilised.	As a deep learning classifier, the extreme gradient boosting approach is employed.	99% for NSL, 96% for CICIDS-001%, and 92% for CIC_IDS2017 are experimental results.	Because deep learning algorithms are used to separate and disseminate regular or suspect network traffic attacks, measuring complexity time has taken many hours.

## Proposed model

**Fig. 1:**
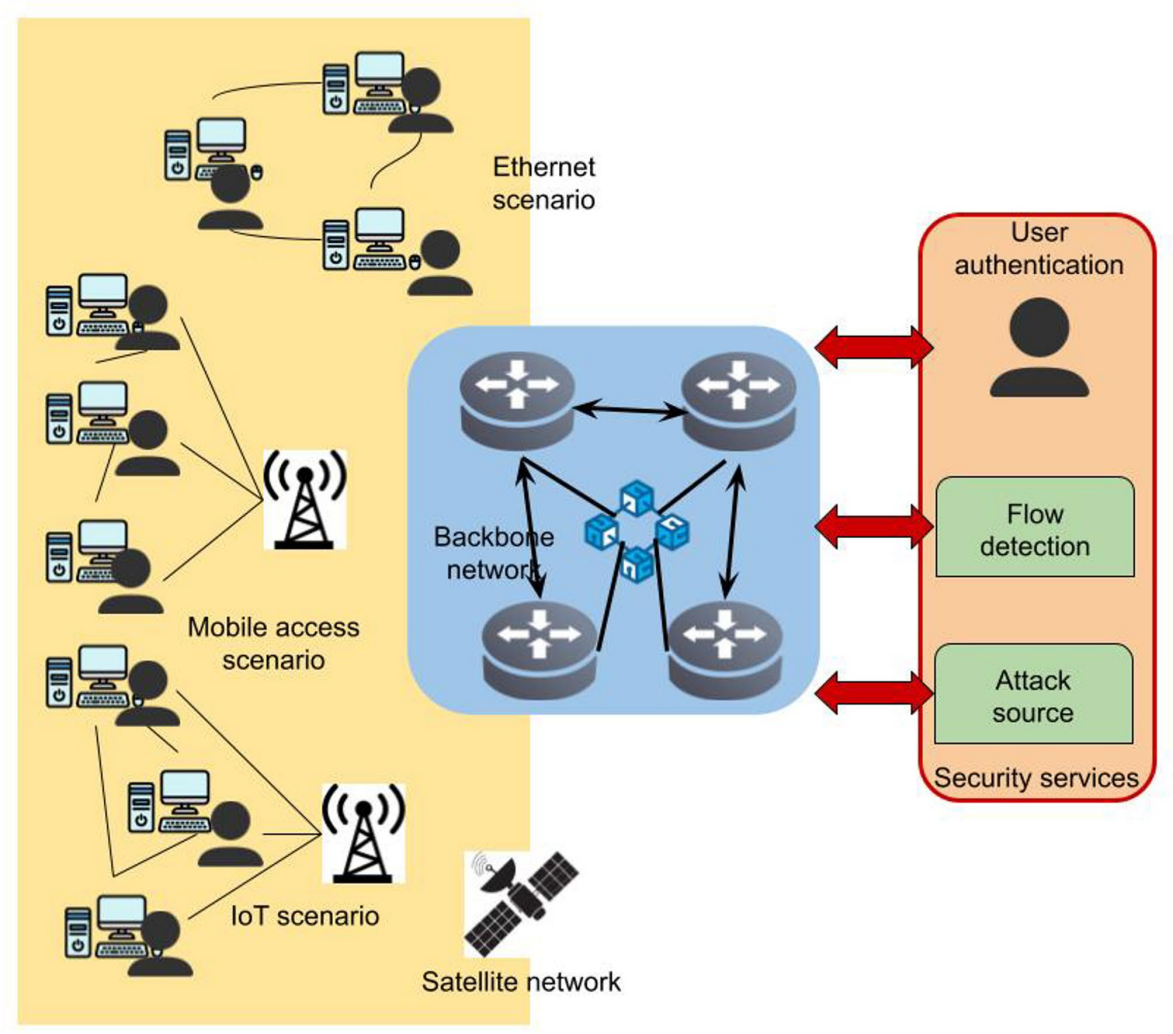
Proposed intrusion detection and cyberattack monitoring method for 6 g networks.

Deployment of the model: As shown in Fig. 1, we have envisioned scenarios for 6G mass access devices with Ethernet, mobile, IoT, and satellite networking. After that, we executed a blockchain-based incarnation of Federated Learning (FL) for the obscurity behaviour database of distributed network security in every 4 high-performance access gateways deployed in spine network. The security architecture we proposed, and AI technologies can provide multiple security services, such as user verification, flow monitoring, identifying the source of an attack.

### Data preprocessing and sampling

First, the data was examined for inconsistencies, mistakes, and duplicate values. Encoding its numerical representation in the Bytes column was 1 K instead of 1000, and the entries in the Flows column were the same for every item in the dataset. These were among the few inconsistencies that were discovered. As a result, the Flows column and three other columns about the labels that were not the focus of this study were removed. These columns were Class, AttackID, and AttackDescription. Since primary goal of this study is to calculate different ML as well as DL^36^ for attack identification and definition, only AttackType was taken into account. Other changes were also made to address the issues with the Bytes column. In particular, for instance, “K” was replaced for 103 and “M” for 106, and then the proper numeric form of resultant match was availed. The data was organized sequentially based on the “Date first seen” feature^37^ to maintain correct order. Following these procedures, three numerical features and seven categorical features were retained. The resulting feature vector consisted of features: Bytes, Flags, Src IP, Src Port, Duration, ToS, Proto, Dest IP, Dest Port, and Packets. Ordinal encoding leverages the use of an imported object, which is an array of integers, in order to encode all the properties, including Src IP and Dst IP, which are not numerical to a number size format because it expected that the value provided as input for the algorithms applies a numerical matrix. Lastly, to improve the effectiveness of the aforementioned methods, each feature was adjusted using min-max normalization between 0 and 1. Min-max scales use the following formula to examine each feature separately by Eq. 1.

1 $$\:{x}^{{\prime\:}}=\frac{\left(x-{x}_{min}\right)}{\left({x}_{max}-{x}_{min}\right)}$$

Given the vast number of fluxes, the successful analysis of this study demands considerable hardware. This problem was resolved by limiting the scope and using only a portion of the data, significantly mitigating memory, processing power^38^, and time requirements. The employment of stratified and random split strategies was not feasible and would not maintain the flow sequence, which is one of the goals of this study, which aims to comprehend how temporal dependencies impact intelligent attack detection and categorization^39,40^. Table 2 compares the selected sample, which includes first two weeks of OpenStack environment, with entire dataset regarding size and class distribution.

**Table 2:** Comparison of attack type class distribution.

Class	Dataset	OpenStack 1 st Half	Sample
Total Records	$\:\text{32,730,224}\left(100\%\right)$	$\:\text{18,772,353}\left(100\%\right)$	$\:\text{2,525,556}\left(100\%\right)$
No Attack	$\:\text{29,452,263}\left(89.95\%\right)$	$\:\text{15,626,326}\left(82.75\%\right)$	$\:\text{2,082,551}\left(82.53\%\right)$
Brute Force	$\:9878\left(0.03\%\right)$	$\:4982\left(0.03\%\right)$	$\:1252\left(0.05\%\right)$
DoS	$\:\text{2,859,227}\left(9.07\%\right)$	$\:\text{2,859,627}\left(15.77\%\right)$	$\:\text{398,470}\left(15.40\%\right)$
Port Scan	$\:\text{304,358}\left(0.93\%\right)$	$\:\text{264,917}\left(1.41\%\right)$	$\:\text{50,135}\left(1.98\%\right)$
Ping Scan	$\:6096\left(0.02\%\right)$	$\:6096\left(0.03\%\right)$	$\:1067\left(0.04\%\right)$

Encoding and data normalization techniques are performed. To make the data convenient, encoded data is reduced in dimensionality. Thus, features are improved to yield the best data features. This is important when looking for anomalies in the data processed. In the subsequent preprocessing stages, the cleaned and organized data advances to the next level, where the Chi-square test is employed to focus on the probabilities of characteristics influencing the outcomes. Eventually, the developed methodology will identify and predict malicious activity on network traffic using the proposed models as a classifier engine. It comprises several phases, each with a designated purpose and several tasks. Each stage’s outcome is the following stage’s input. The phases are explained systematically after one another.

### Blockchain federated Gaussian multi-agent Q- encoder neural networks (BFGMAQENN)

The FL configuration includes local models and distributed smart factory nodes. Unlike a centralised learning environment, K smart factories study a local approach in a FL fashion. Despite having same structure, the k = 1…, K local methods are trained using various datasets from their connected clients. The FL method is launched by the parameter server at t = 0, and the starting weights for the local methods are specified. Parameter server is then used to download these local models to each of the k = 1…, K smart factories. Third, each of the E = 1…, E local methods use training data from matching blockchain datasets to evaluate a new local set of weights in parallel. Parameter server employs a technique of weighted averaging to merge the weights produced by every client’s local method yielding an improved global method in turn. Up to when a certain stop criterion is reached, every time cycle goes back, a new epoch commences. The acquired network data from a blockchain’s network can be utilized to periodically update the training data. This training data can also be labelled by means of several processes such as expert evaluation, user feedback systems, automatic threat feeds that include honeypots, security alerts, misleading environments and even cryptographic validation. At iteration i, we refer to Di n as the dataset of cluster n, where n ∈ {0…, N}. Additionally, a deep learning model that functions as the BFGMAQENN model is present in each cluster. The following formula can be used to determine the deep learning model’s output in a cluster n at iteration I by Eq. 2.

2 $$\:{I}_{n}^{i}={H}_{n}{D}_{n}^{i}$$

where Hn is a transfer function of the CCD model in cluster n and I i n is the output prediction at iteration i of the CCD model in cluster n. We represent L (·) as the BFGMAQENN model’s loss function and Y n as the vector of labels corresponding to Dn. ∇L (θ i n; Di n) is the notation for the gradient (trained model) ∇θ i n of the deep model at cluster n at iteration i. The CCD model’s weights can then be updated in the manner described below (3)

3 $$\:{\theta\:}_{n}^{i}={\theta\:}_{n}^{i-1}-\mu\:{\nabla\:}_{{\theta\:}_{n}}L\left({\theta\:}_{n};{D}_{n}^{i}\right)$$

where ∇θn L (θn; Di n) is gradient of L (·) with respect to θn calculated for Di n, and µ is the learning rate. The following is the clipping of the local updates △θ i n = θ i n – θ i − 1 n to a maximum norm ϑ by Eq. 4.

4 $$\:\varDelta\:{\theta\:}_{n}^{i}\leftarrow\:\varDelta\:{\theta\:}_{n}^{i}min\left(1,\frac{\vartheta\:}{\parallel\:\varDelta\:{\theta\:}_{n}^{i}\parallel\:}\right)$$

The learning process is stabilized by the clipping, which limits the impact of any one update. We added a divergence estimation method to improve the BFGMAQENN. As a result, method is more accurate and resilient to hostile attacks.

### Divergence estimation

Determining the difference and disparity between every client’s data distribution and global model distribution is known as divergence estimation. It offers information about how representative the data from each client is about the global model’s overall data distribution. We employed the Kullback–Leibler (KL) divergence to estimate divergence in Federated Learning situations. The KL divergence quantifies data lost when utilizing one probability distribution to approximate another and calculates relative entropy between the two. Equation 5 is used to express it.

5 $$\:{D}_{KL}\left({P}_{i}\left|\right|{P}_{t}\right)=\sum\:_{i}{P}_{i}log\frac{{P}_{i}i}{{P}_{t}i}\:\:$$

KL divergence between local method Pi as well as global method Pt is determined in this case by this equation. The total is the sum of all potential outcomes.

### Weight calculation

The next step is determining important weights for every client after estimating the divergence between the global model distribution and every client’s data distribution. The importance or applicability of each client’s data about the global method is reflected in these weights. We determined the important weight by taking the inverse of the calculated divergence. With this method, clients with data distributions that are closer to the global method will contribute more to the aggregation process than clients with data distributions that are more dissimilar. We outline the most popular frameworks for simulating these apps’ environments. The concept is extended to many agents after starting with the single-agent formulation. Finding accurate estimations of state and/or state–action pair value functions ()V(s) and (, ) Q (s, a) is the aim of value-based approaches. A fixed rule maps the value functions to actions and chooses the best policy. For instance, the − policy chooses action with a greater Q-value with probability 1− and a random action with probability ϵ. We can obtain formula if we take the reward, for instance, as the goal function by Eq. 6.

6 $$\:\:{\nabla\:}_{\theta\:}E\left[R\right(S,A\left)\right]=E\left[{\nabla\:}_{\theta\:}log{\pi\:}_{\theta\:}(A\mid\:S)R(S,A)\right]$$

This concept is known as the policy gradient theorem. It enables gradient ascent by allowing the policy to differentiate, even when the expected reward value cannot be directly differentiated. Q-learning agent must choose the best course of action to maximize total discounted reward; this course of action, ∗, is such that by Eq. 7.

7 $$\:{V}^{\text{*}}\left(s\right)\equiv\:{V}^{{\pi\:}^{\text{*}}}\left(s\right)=\underset{a}{max}\:\left\{{R}_{s}\left(a\right)+\gamma\:\sum\:_{{s}^{{\prime\:}}}{P}_{s{s}^{{\prime\:}}}\left[a\right]{V}^{{\pi\:}^{\text{*}}}\left({s}^{{\prime\:}}\right)\right\}$$

By constructing a matrix (, ) of dimensions × that contains evaluates of all the value functions at time-step n, goal is to evaluate state–action pair value function. Q-values are changed utilizing equation at each learning iteration n by Eq. 8.

8 $$\:{Q}_{n}(s,a)=\left\{\begin{array}{c}\left(1-{\alpha\:}_{n}\right){Q}_{n-1}\left(s,a\right)+{\alpha\:}_{n}\left[{r}_{n}\text{*}\gamma\:{V}_{n-1}\left({s}_{n+1}\right)\right]\:\:\:\:\:\:\:\:\:\:\:\:\:if\:s={s}_{n}\\\:and\\\:\:{a}_{n}\:{Q}_{n-a}(s,a)\:\:\:\:\:\:\:\:\:\:\:\:\:\:\:\:\:\:\:\:\:\:\:\:\:\:\:\:\:\:\:\:\:\:\:\:\:\:\:\:\:\:\:\:\:\:\:\:\:\:\:\:\:\:\:\:\:\:\:\:\:\:\:\:\:otherwise\:\:\:\end{array}\right.\:$$

$\:\:{V}_{n-1}\left({s}_{n+1}\right)=\underset{{a}^{{\prime\:}}}{max}\:{Q}_{n-1}\left({s}_{n+1},{a}^{{\prime\:}}\right)$ under assumption of bounded rewards ||≤| and learning rates 0≤<1 such that by Eq. 9.

9 $$\:\:{\sum\:}_{i=1}^{\infty\:}{\alpha\:}_{{n}^{{\prime\:}}(s,)}\:\:=\infty\:,{\sum\:}_{i=1}^{\infty\:}{\left[{\alpha\:}_{{n}^{{\prime\:}}(s,a)}\right]}^{2}\:\:<\infty\:\:\:\forall\:s,a,$$

With probability 1, evaluates (, ) will converge to ideal Q-value ∗ (, ). A typical convolution layer with eight filters is the first layer in the encoding step. The series of leftover blocks with an increasing number of channels comes next. The suggested efficient deconvolution (EF-deconvolution) layer is used to up-sample the feature map at each stage of the decoding module. This layer can execute linear modification to provide richer features and filter out a more meaningful set. The generated feature map is fused with the feature map corresponding to the encoding module’s output. The first EF-deconvolution layer is introduced with features that are the same size as the encoding module. This is comparable to feature augmentation; the following three EF-deconvolution layers are connected to enrich the features. The above process is explained in the BFGMAQENN algorithm.

**Algorithm 1:**
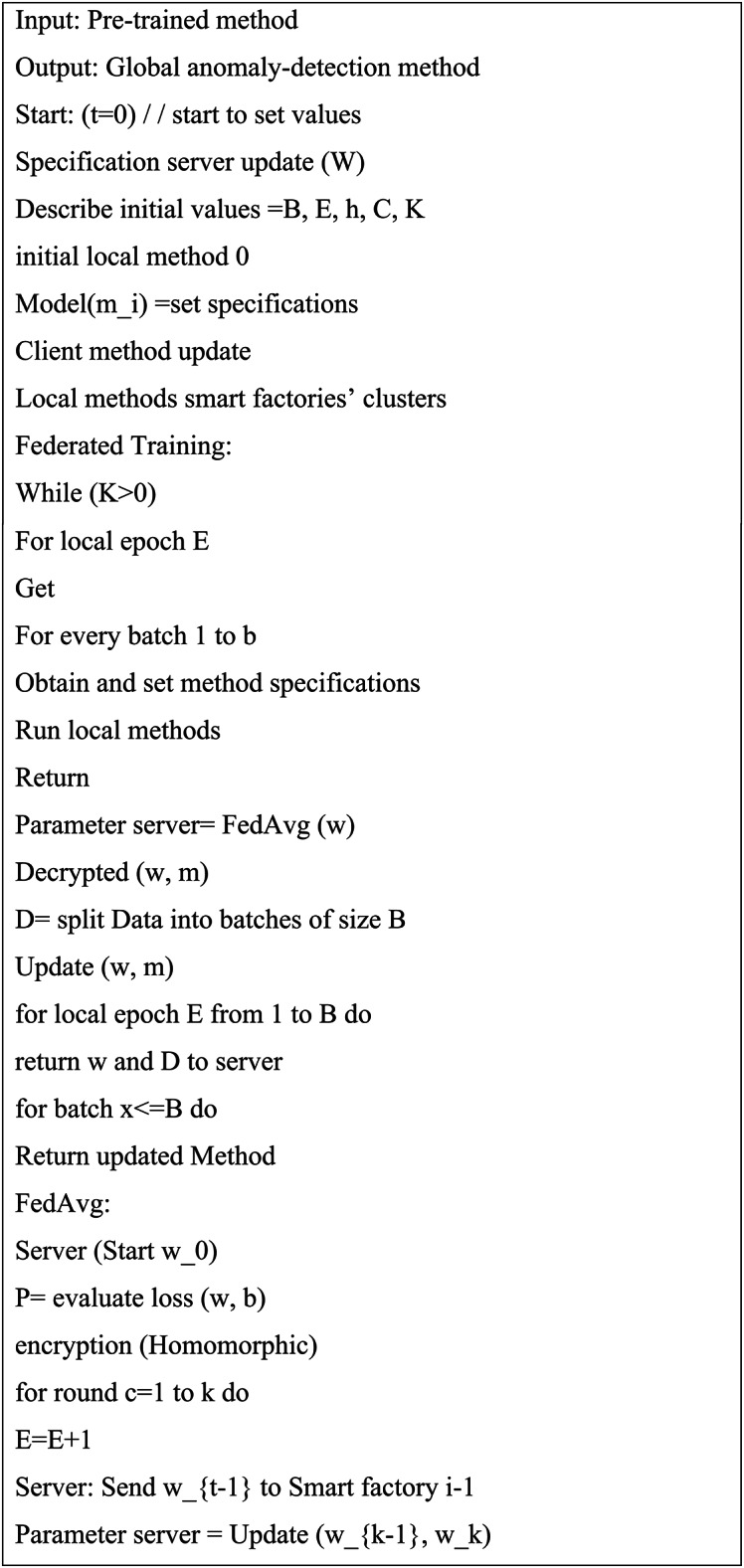
Algorithm of Blockchain Federated Gaussian Multi-Agent Q-Encoder Neural Networks (BFGMAQENN).

### Whale swarm binary Wolf optimization (WSBWO)

**Fig. 2:**
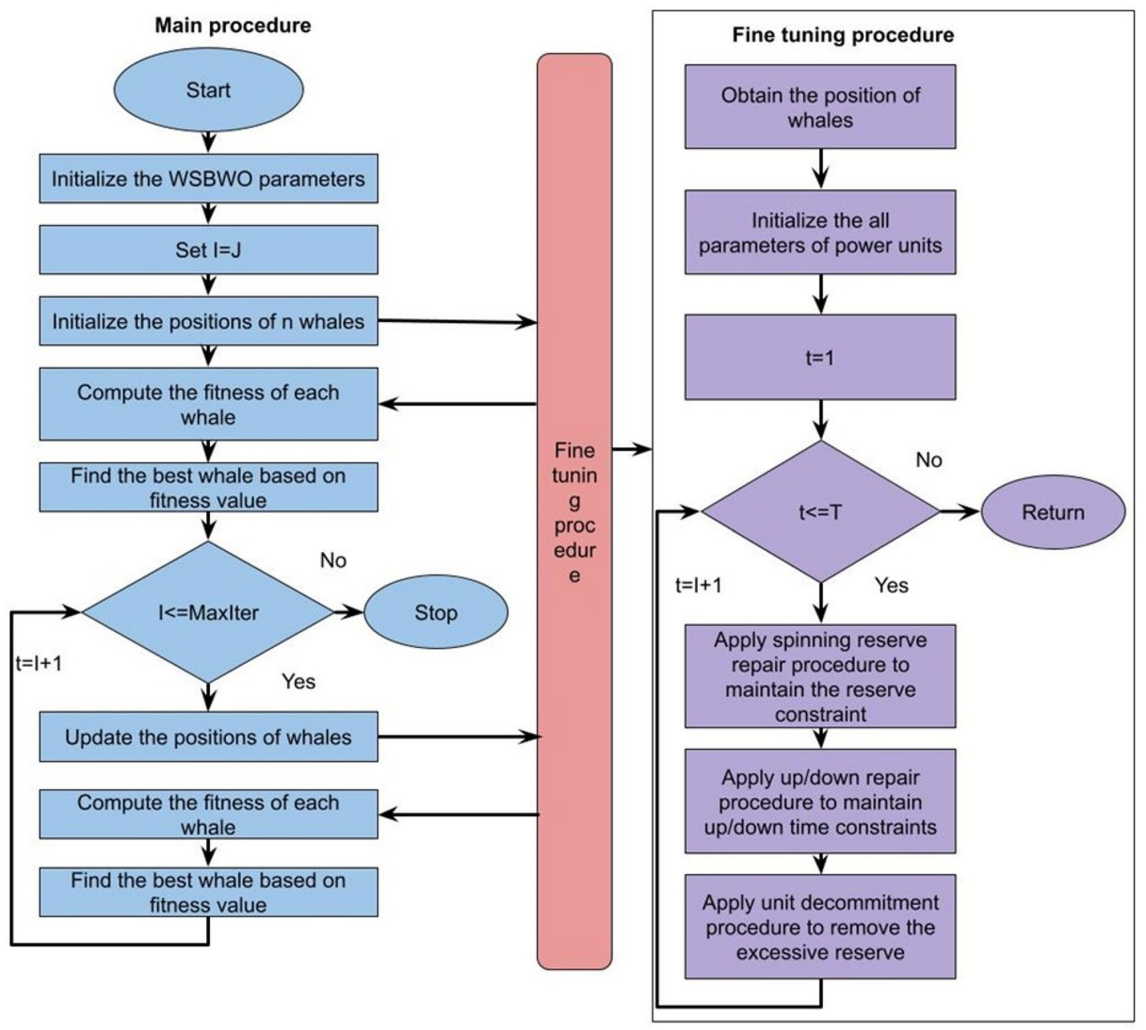
Whale Swarm Binary Wolf Optimization Algorithm.

To develop algorithms inspired by whale swarms, it has idealized several whale-hunting rules. To make it easier to understand our most recent Whale Swarm algorithm, the following four idealized concepts are applied as shown in Fig. 2:

### All whales capable of coordinating with one another through the search area

2) The existence of each whale with the statistical ability to determine how far away the whales of the other group are.

3) the kind of consumption and its weight that are more relevant to the related whale 4) The behaviour of the whale. The following formula is used by WSA to update a whale X’s position based on its “better and nearest” whale Y by Eq. 10.

10 $$\:{x}_{i}^{t+1}={x}_{i}^{{\prime\:}}+rand\left(0,{\rho\:}_{0}\cdot\:{e}^{-\eta\:\cdot\:dd,y}\right)\text{*}\left({y}_{i}^{{\prime\:}}-{x}_{i}^{t}\right)$$

where x t + 1 and x t i are i-th element of X’s position at iteration t is represented by i, while i-th element of Y’s position at iteration t is represented by y t i. The ultrasonic source’s intensity, ρ0, can be set to 2 in practically all situations. The natural constant is represented by e. The attenuation coefficient is denoted by η. Equation 10 states that a whale would move randomly and modelled when guided by its “better and nearest” whale, which is located nearby, randomly and negatively when guided by that whale, which is located relatively far away. According to Eq. 10, every whale in a community tends to follow the positive and random movements of a nearby “better and nearest” whale.

The solution representation above shows that every element is spaced about others by measuring the binary numerical differences. Both the first and second layers of every solution have n components. Using two comparable replies, determine whether two things in the exact location are distinct. If two elements are the same, the distance between their two places is zero; otherwise, it is one. The total distance of all the related components between two solutions is the distance between them. It is believed that one of the two randomly chosen individuals from the parent population, C, will stay frozen while the parent population is searched for C’s closest relative, R. The method above determines the distance between the two people. Additionally, the crossover operator considers the closest individual to R as the second individual. Grey wolf’s social structure and hunting strategy served as the algorithm’s model. Initially, when creating GWO, four wolves, or tiers of social hierarchy, are explained as follows: • Answer with highest objective value is alpha (α) wolf. • Solution with second-best objective value is eta (β) wolf. • Solution with third-best objective value is delta (δ) wolf. • Omega (ω) wolf: every alternative remedy algorithm’s hunting mechanism is guided by first three suitable wolves, α, β, and δ. The other wolves follow them and regarded as ω. Grey wolves hunt in a series of well-planned phases, including encircling, hunting, and attacking. Encircling process of gray wolves was quantitatively modelled using the following formulas Eqs. 11 and 12.

$$\:\overrightarrow{D}=\left|\overrightarrow{C}\cdot\:{\overrightarrow{X}}_{p}\left(t\right)-\overrightarrow{X}\left(t\right)\right|$$

11 $$\:\:\overrightarrow{X}(t+1)={\overrightarrow{X}}_{p}\left(t\right)+\overrightarrow{A}\cdot\:\overrightarrow{D}$$

$$\:\overrightarrow{A}=2\overrightarrow{a}\cdot\:\overrightarrow{{r}_{1}}-\overrightarrow{a}\:$$

12 $$\:\overrightarrow{C}=2\cdot\:\overrightarrow{{r}_{2}}$$

where the random vectors $\:\overrightarrow{{r}_{1}}$ and $\:\overrightarrow{{r}_{2}}$ are in [0,1]. In contrast, $\:\overrightarrow{a}\:$elements decrease linearly from 2 to 0 iterations. Additionally, GWO maintains initial 3 best solutions (α, β, and δ) that have been found thus far and requires ω wolves to adjust their placements with relation to these. Therefore, a series of equations are continually performed for each search candidate in order to replicate grey wolf’s hunting method by Eq. 13.

13 $$\:\overrightarrow{X}(t+1)=\frac{\overrightarrow{{X}_{1}}+\overrightarrow{{X}_{2}}+\overrightarrow{{X}_{3}}}{3}$$

A vector $\:\overrightarrow{a}$, which is a random vector with elements in range [− a, a], can be used to describe the grey wolfs’ attacking mechanism. Elements of $\:\overrightarrow{a}$ fall linearly from 2 to 0 with each repetition, this is expressed as follows Eq. 14

14 $$\:\overrightarrow{a}=2-t\cdot\:\frac{2}{\:maxIter\:}$$

where t is iteration timer and maxIter is maximum number of iterations that GWO can employ to evaluate global optimum. Two primary components of modified GWO are (1) making more wolves rather than wolves and (2) putting forth a new fitness function that seeks to ascertain whether or not a subgroup of wolves meets the key objectives. When proposing the fitness function to calculate every subset of the selected features^42^, the significance of accuracy and the total number of selected characteristics are considered. Therefore, the prediction system’s overall performance is determined by how well it predicts unknown classes in terms of accuracy and total number of features chosen^43^. For this situation, the conventional continuous version of an optimizer needs to be adjusted^44^. Here, a method for converting the suggested optimizer’s continuous values to binary values is described as Eq. 15.

15 $$\:{\overrightarrow{G}}_{d}^{\left(t+1\right)}=f\left(x\right)=\left\{\begin{array}{c}\:\:\:\:\:\:\:\:1\:\:\:\:\:\:\:\:if\:Sigmoid\:\left(x\right)\ge\:0.5\:0\\\:otherwise\\\:\:\text{Sigmoid}\left(x\right)=\frac{1}{1+{exp}^{-10\left(x-0.5\right)}}\\\:x=\frac{\overrightarrow{{G}_{\alpha\:}^{{\prime\:}}}\overrightarrow{{G}_{1}^{{\prime\:}}}+\overrightarrow{{G}_{\beta\:}}\overrightarrow{{G}_{2}}+\overrightarrow{{G}_{\delta\:}}\overrightarrow{{G}_{3}^{{\prime\:}}}}{\overrightarrow{{G}_{\alpha\:}^{{\prime\:}}}+\overrightarrow{{G}_{\beta\:}}+\overrightarrow{{G}_{\delta\:}}}\end{array}\right.$$

The second and third best individuals are $\:\overrightarrow{{G}_{\beta\:}}$ and $\:\overrightarrow{{G}_{\delta\:}}$ In Eq. 15, updated positions are $\:\overrightarrow{{G}_{\text{0,1}}}$, $\:\overrightarrow{{G}_{\text{0,2}}}$ and $\:\overrightarrow{{G}_{\text{0,3}}}$. Sigmoid function is responsible for scaling continuous values to either 0 or 1. To determine whether the dimension will be zero or one, the Sigmoid(x) > 0.5 condition is applied.

## Results and discussion

Table 3 illustrates the system configuration for the proposed approach, which comprises a Windows 10 computer equipped with an Intel Core i5-8300 H CPU operating at 2.3 GHz, a GeForce GTX 1050 graphics card, and 8 GB of RAM, along with the specified parameters. The Python Software Foundation created Python 3.6, and NVIDIA Corporation developed CUDA 10.2.

**Table 3:** Simulation setting of the proposed Model.

Component	Specification
**Operating System**	Windows 10
**Processor**	Intel Core i5-8300 H (2.3 GHz)
**GPU**	NVIDIA GeForce GTX 1050
**RAM**	8 GB
**Programming Language**	Python 3.6 (Python Software Foundation)
**CUDA Version**	CUDA 10.2 (NVIDIA Corporation)

### Cyberattack dataset description

Coburg Intrusion Detection Dataset (CIDDS-001)^1^ revealed approximately four weeks of network traffic from 5:43:57 p.m. on March 3, 2017, to 11:59:30 p.m. on April 18, 2017. This dataset includes approximately 33 million flows recorded from two separate environments: an external server capturing accurate and current internet traffic and a simulated small business environment. Several clients and standard servers, such as email or web servers, are part of the OpenStack ecosystem. In addition to regular activity, the dataset includes labelled flow-based data to assess anomaly-based network IDS against DoS, Brute Force, Ping, and Port Scan attacks. A GitHub repository hosts the Python programs used for traffic generation. The CIDDS-001 dataset, being relatively recent, provides an extensive collection of network flows and includes a wide range of contemporary attack types. This makes it a highly reliable resource for researching and evaluating network-based intrusion detection techniques. Its comprehensive coverage of modern attack scenarios contributes to its effectiveness in assessing and improving cybersecurity methods. A unidirectional Netflow format describes the data collection made available by the CIDDS-001 dataset.

DARPA (Defense Advanced Research Project Agency) created first IDS dataset^2^ using the KDD98 dataset. DARPA launched a program at MIT Lincoln Labs in 1998 to provide a comprehensive and realistic IDS benchmarking environment (MIT, 1999). The 41 attributes in the KDD training dataset are separated into two groups: attack and normal. There will be just one kind of attack. These datasets need to be updated and do not contain data on recent malware outbreaks. KDD99 is still widely used by scholars and is regarded as a benchmark in the IDS research community. CAIDA^3^ This collection includes network activity records from Distributed Denial-of-Service (DDOS) attacks. DOS attack aims to interfere with regular traffic and stop legitimate communication from getting to its intended location by overloading a computer or network with network packets. Attack diversity is one of the CAIDA dataset’s shortcomings. Furthermore, distinguishing between aberrant and typical traffic flows is challenging because network-wide features are not included in the collected data. KYOTO Features for IDS network analysis and evaluation are included in the Kyoto dataset, including 14 data samples from KDD Cup ‘99 dataset. The Kyoto 2006 + dataset^4^ examined darknet data obtained on a variety of real as well as virtual honeypots and honeypots that were not utilized as honeypots. It was gathered utilizing honeypots, darknet sensors, an email server, and a web crawler. There are 50,033,015 routine sessions, 43,043,225 assault sessions, 425,719 sessions associated with abnormal behaviour during the monitoring period. This dataset eliminates specific intrusion data and prevents problems like duplicate complaints.

### Simulation parameter analysis

Accuracy:

16 $$\:Accuracy=\frac{(TP+TN)}{\left(TP+TN+FP+FN\right)\text{*}100}$$

where,

•TP—True positive.
•TN—True negative.
•FP—False positive.
•FN—False negative.

Data integrity: A data integrity formula for a data file is σi = (H(mi) ⋅ umi) α, where:

- **H**: A cryptographic hash function.
- **u**: A random number.
- **α**: A system master secret defined on the integer field being used.

### Scalability

The following is a summary of the scalability parameters: As a function of load (N), calculate the throughput X(N). It is OK to use a sparse data sample (more than four loads). Using the data, determine the efficiency C/N, the capacity ratio C(N), and its inverse N/C.

The formula to calculate the total delay in communication overhead is:

17 $$\:\left(n-2\right)l+s/b$$

Where:

: is the number of messages sent.

ℓ: is the latency in seconds.

: is the total size of all messages.

: is the bandwidth in bytes per second.

### Security based result analysis

Table 4 presents a comparative analysis of various cyberattack datasets used in intrusion detection systems. The datasets evaluated include CIDDS-001, KDD98, CAIDA, and KYOTO. These datasets are analysed based on several key criteria: detection accuracy, data integrity, scalability, communication overhead, and network efficiency.

**Table 4:** Proposed model analysis for various intrusion detection based cyberattack Dataset.

Dataset	Techniques	Detection accuracy	Data integrity	Scalability	Communication overhead	Network efficiency
**CIDDS-001**	**LA-HLRW**	70	68	72	75	78
	**SSHA**	74	72	75	78	81
	**BFGMAQENN_WSBWO**	80	79	78	65	83
**KDD98**	**LA-HLRW**	71	73	74	70	75
	**SSHA**	73	78	77	79	83
	**BFGMAQENN_WSBWO**	87	84	81	67	86
**CAIDA**	**LA-HLRW**	73	70	76	77	83
	**SSHA**	80	84	79	83	86
	**BFGMAQENN_WSBWO**	89	90	84	62	91
**KYOTO**	**LA-HLRW**	81	82	84	79	85
	**SSHA**	84	85	88	89	88
	**BFGMAQENN_WSBWO**	97	94	93	60	98

**Fig. 3:**
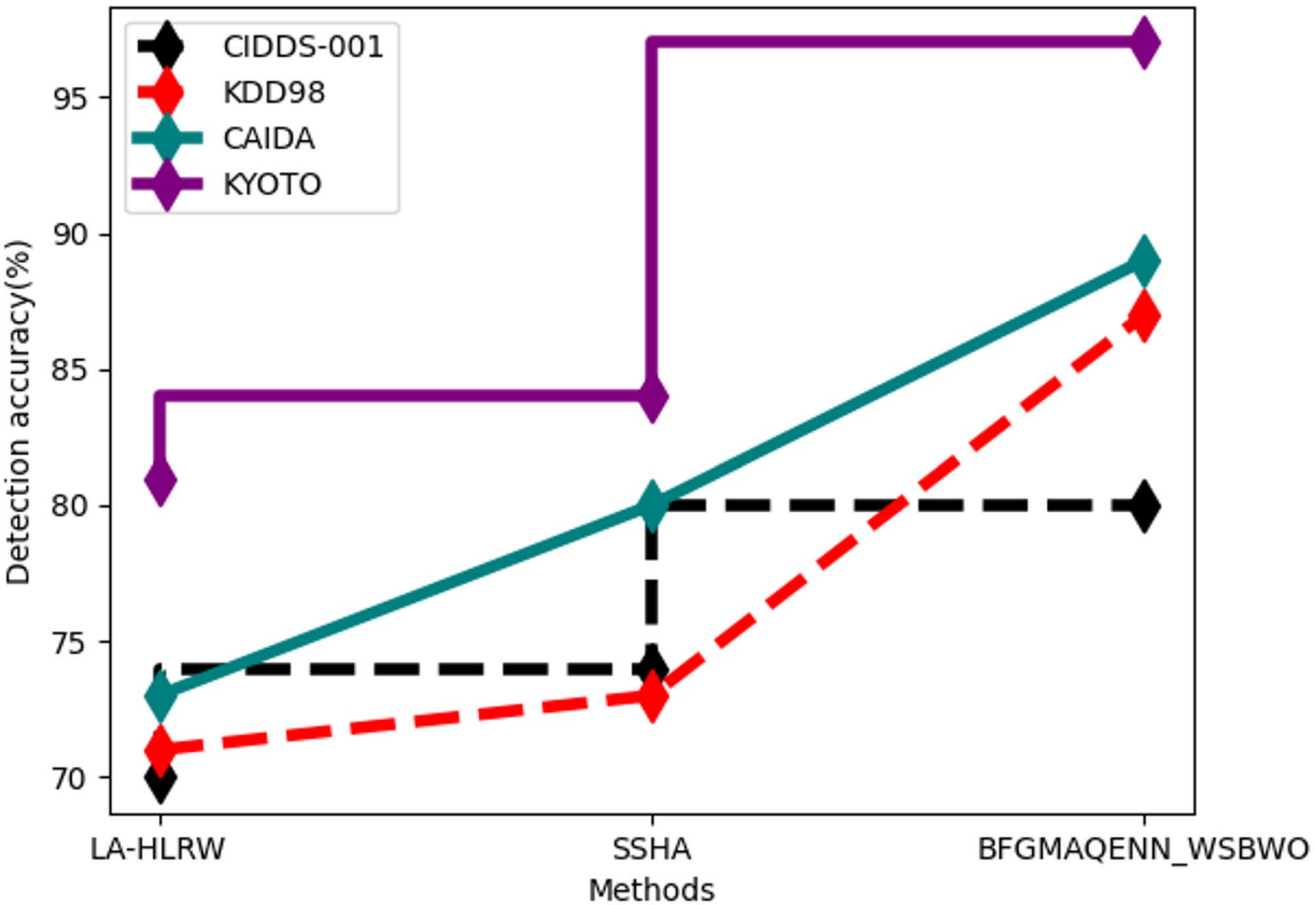
Comparison of detection accuracy.

Figure 3 illustrates the detection accuracy comparison. For the CIDDS-001 dataset, the proposed technique achieved an 80% detection accuracy, compared to 70% for the existing LA-HLRW and 74% for SSHA. For the KDD98 dataset, the proposed technique attained 87% detection accuracy, while LA-HLRW achieved 71% and SSHA 73%. With the CAIDA dataset, the proposed method reached 89% detection accuracy, outperforming LA-HLRW at 73% and SSHA at 80%. Finally, the proposed technique achieved 97% detection accuracy for the KYOTO dataset, significantly higher than LA-HLRW at 81% and SSHA at 84%.

**Fig. 4:**
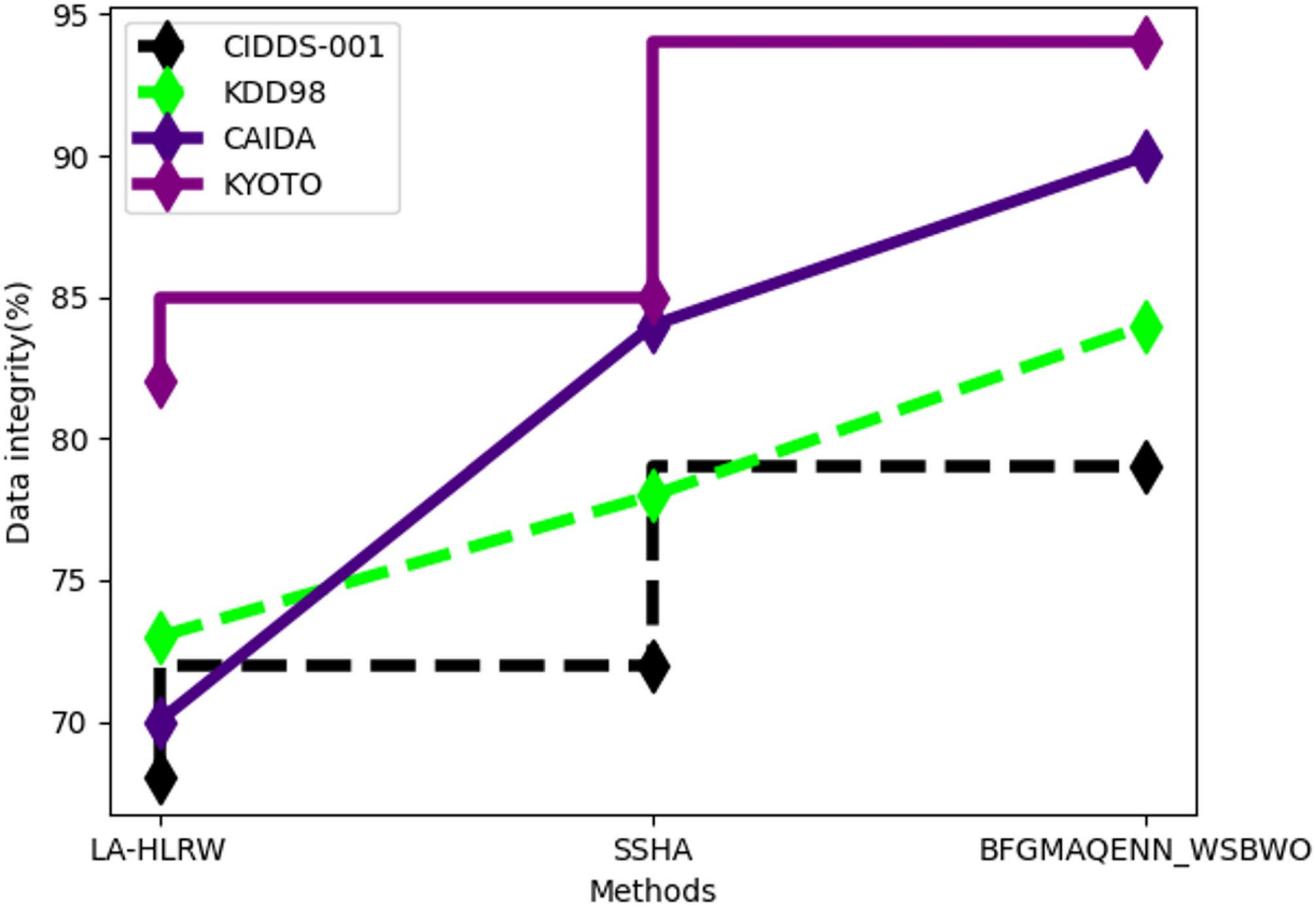
Comparison of Data integrity.

As Fig. 4, presents the analysis of data integrity. For the CIDDS-001 dataset, the proposed technique achieved 79% data integrity, compared to 68% for LA-HLRW and 72% for SSHA. For the KDD98 dataset, the proposed method reached 84% data integrity, outperforming LA-HLRW at 73% and SSHA at 78%. In the CAIDA dataset, the proposed approach recorded 90% data integrity, while LA-HLRW and SSHA achieved 70% and 84%, respectively. Finally, the proposed technique attained 94% data integrity for the KYOTO dataset, surpassing LA-HLRW at 82% and SSHA at 85%.

**Fig. 5:**
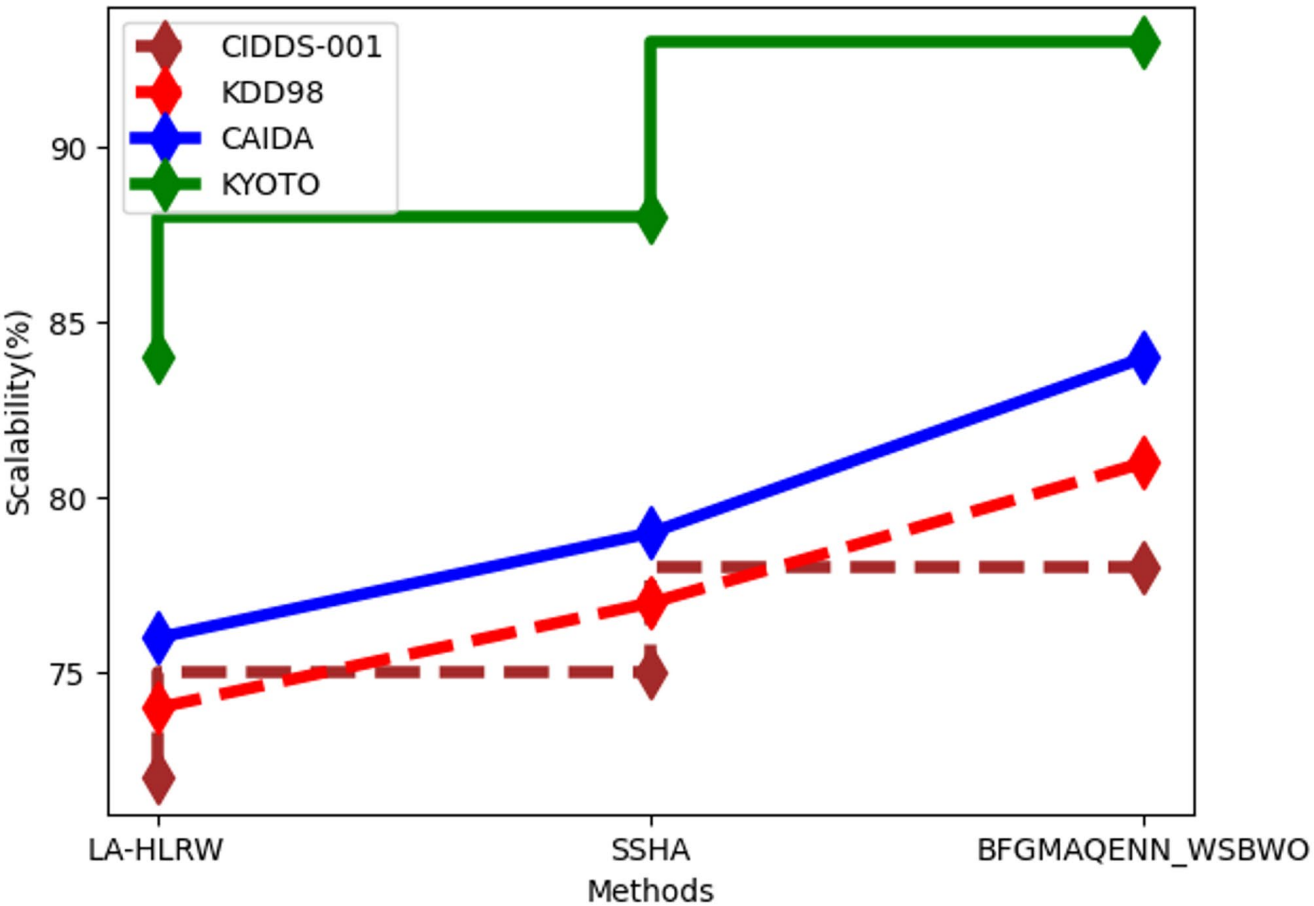
Comparison of scalability.

Figure 5 depicts the scalability analysis. For the CIDDS-001 dataset, the proposed technique achieved a scalability of 78%, compared to 72% for LA-HLRW and 75% for SSHA. In the KDD98 dataset, the proposed method recorded 81% scalability, outperforming LA-HLRW at 74% and SSHA at 77%. For the CAIDA dataset, the proposed approach attained 84% scalability, while LA-HLRW and SSHA achieved 76% and 79%, respectively. Finally, the proposed technique demonstrated 93% scalability for the KYOTO dataset, surpassing LA-HLRW at 84% and SSHA at 88%.

**Fig. 6:**
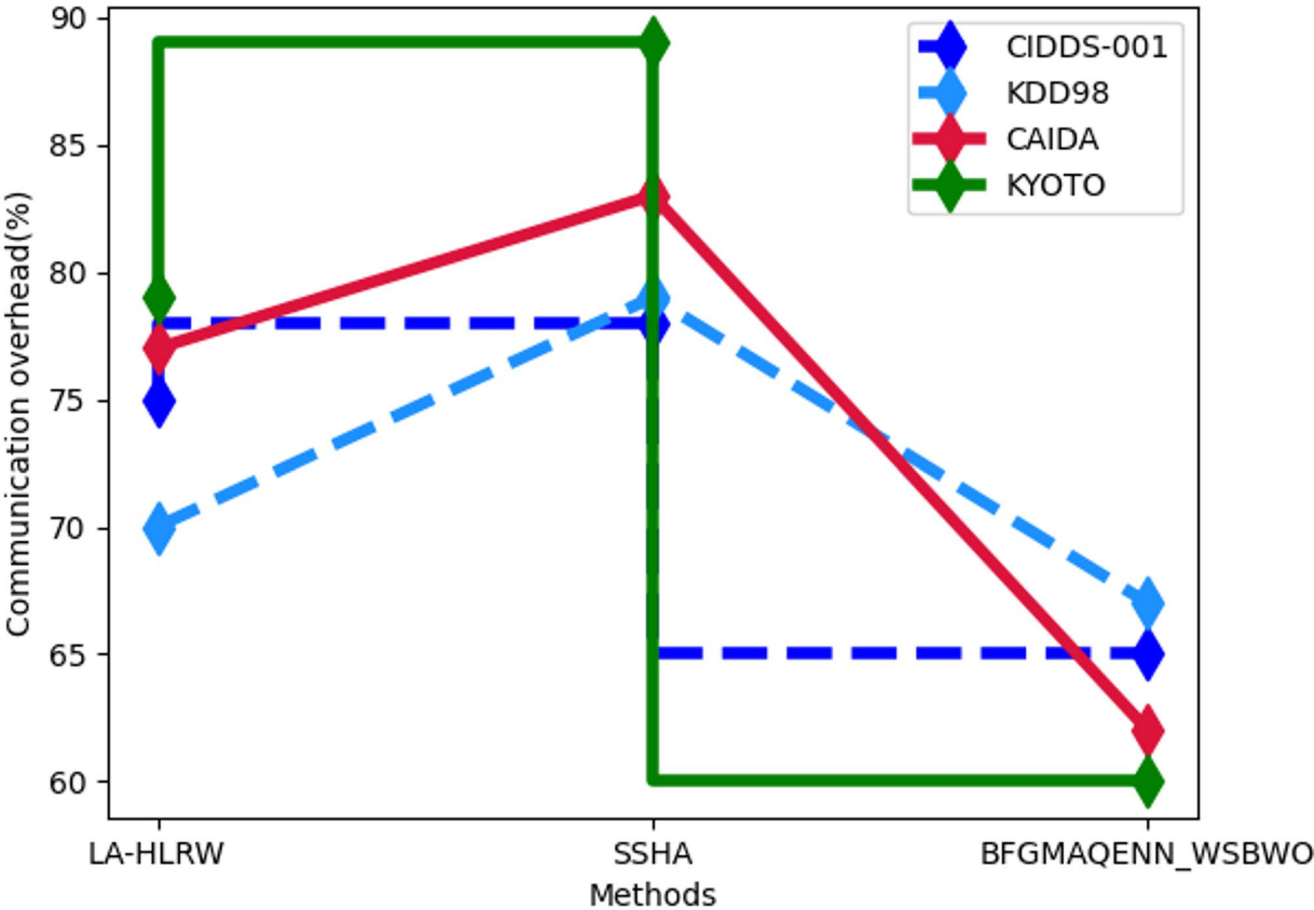
Comparison of Communication Overhead.

Figure 6 presents the analysis of communication overhead. For the CIDDS-001 dataset, the proposed technique achieved a communication overhead of 65%, compared to 75% for LA-HLRW and 78% for SSHA. In the KDD98 dataset, the proposed method recorded 67% communication overhead, while LA-HLRW and SSHA scored 70% and 79%, respectively. For the CAIDA dataset, the proposed approach demonstrated 62% communication overhead, outperforming LA-HLRW at 77% and SSHA at 83%. Finally, the proposed technique achieved 60% communication overhead for the KYOTO dataset, compared to 79% for LA-HLRW and 89% for SSHA.

**Fig. 7:**
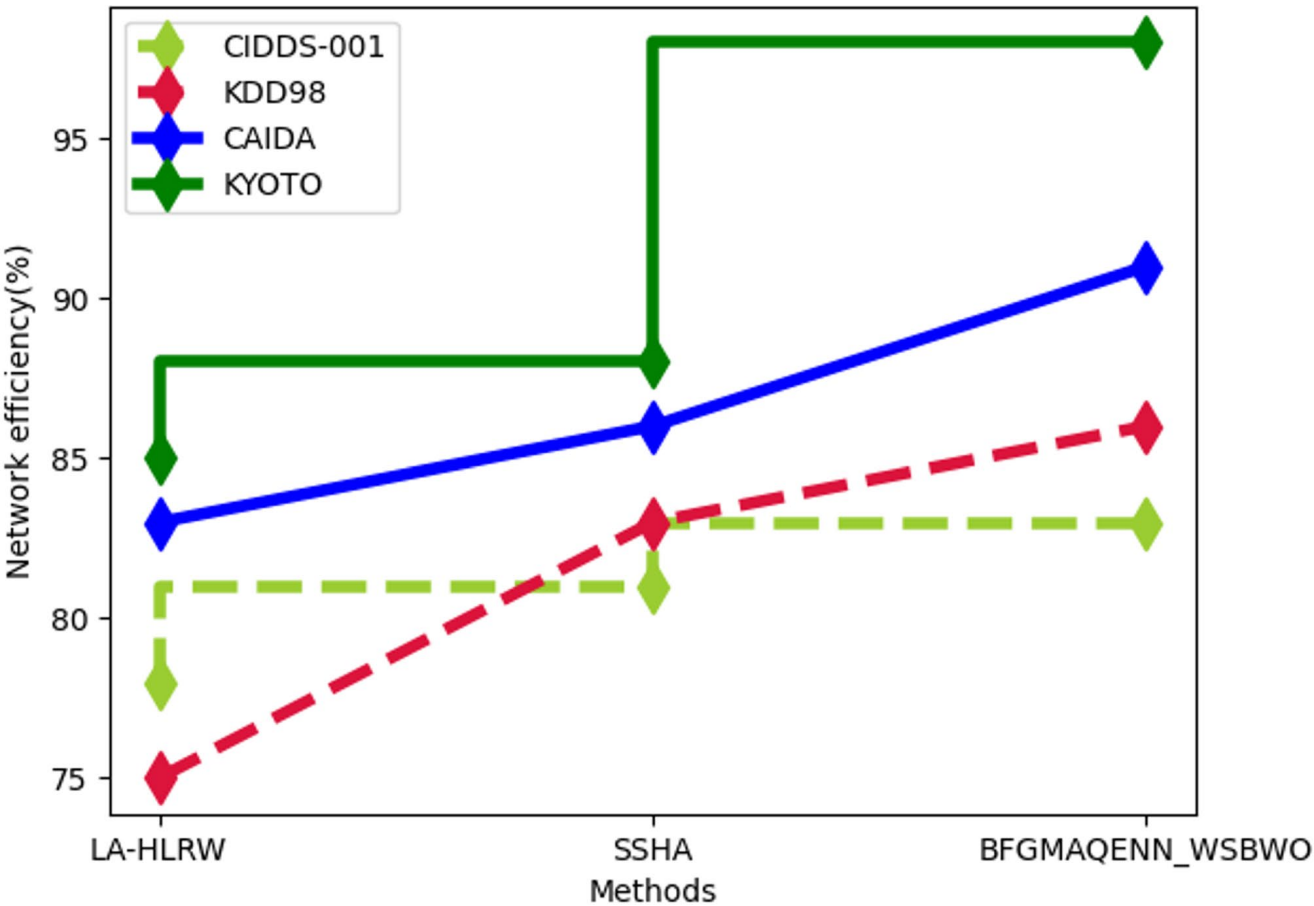
Comparison of Network Efficiency.

Figure 7 highlights the analysis of network efficiency. For the CIDDS-001 dataset, the proposed technique achieved a network efficiency of 83%, surpassing LA-HLRW at 78% and SSHA at 81%. In the KDD98 dataset, the proposed method recorded 86% network efficiency, compared to 75% for LA-HLRW and 83% for SSHA. For the CAIDA dataset, the proposed approach reached 91% network efficiency, outperforming LA-HLRW at 83% and SSHA at 86%. Lastly, the proposed technique achieved 98% network efficiency for the KYOTO dataset, while LA-HLRW and SSHA recorded 85% and 88%, respectively.

**Fig. 8:**
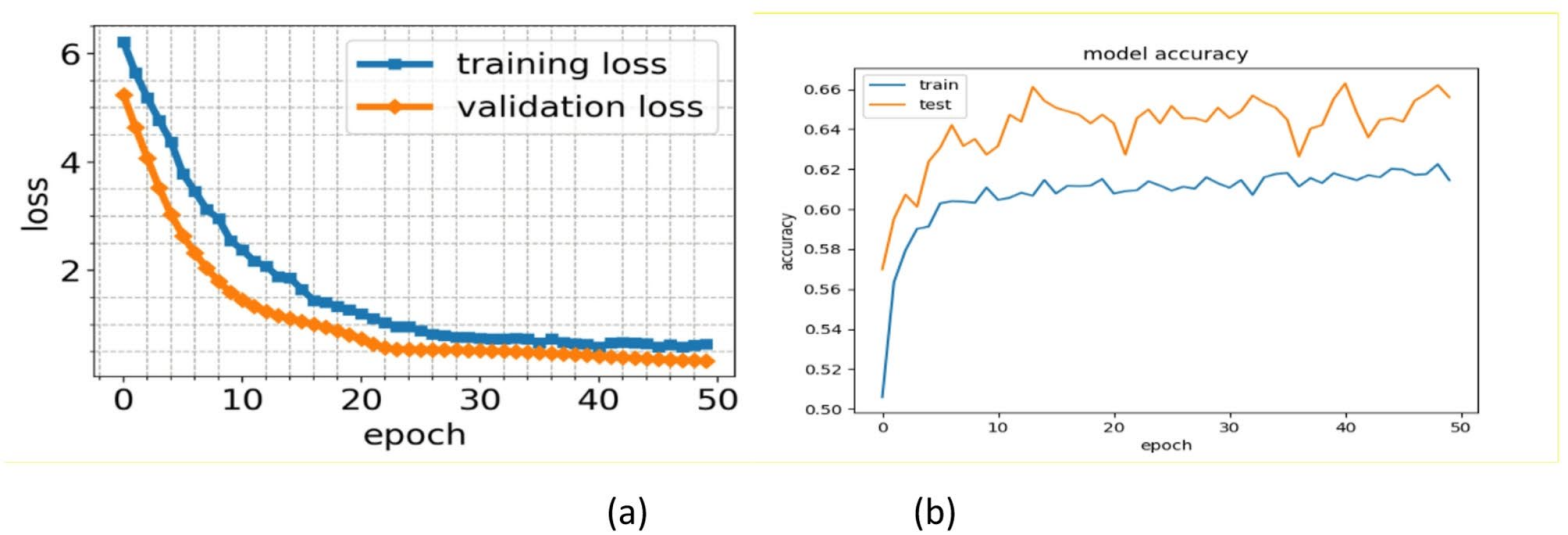
Training and validation learning curves of the proposed model. (**a**) Optimization learning curve., (**b**) Performance learning curve.

The results indicate that proposed method consistently identifies more attacks as flow sequences increase while reducing false-positive rates. This improvement does not favour one class over another, as evidenced by the f1-scores, which either remained stable or improved across all classes. To ensure proper model training, the learning curves in Fig. 8 were analysed in detail. Both training and validation loss values decreased steadily with each epoch, suggesting the absence of underfitting or overfitting. Furthermore, the accuracy scores for both sets were closely aligned and improved progressively as the loss values decreased.

**Table 5:** Accuracy degradation analysis by varying heterogeneity and convergence Lag.

Dataset	Technique	Heterogeneity Level	Detection Accuracy (%)	Accuracy Degradation (%)	Convergence Lag (Rounds)
**CIDDS-001**	LA-HLRW	Low (10%)	78	2	50
		Medium (40%)	74	6	65
		High (80%)	70	10	80
	SSHA	Low	82	2	50
		Medium	78	6	65
		High	74	10	80
	BFGMAQENN_WSBWO	Low	86	1	50
		Medium	83	4	65
		High	80	7	80
**KDD98**	LA-HLRW	Low	80	2	50
		Medium	76	6	65
		High	71	11	80
	SSHA	Low	84	1	50
		Medium	79	6	65
		High	73	12	80
	BFGMAQENN_WSBWO	Low	89	0.5	50
		Medium	87	2.5	65
		High	84	5.5	80
**CAIDA**	LA-HLRW	Low	81	3	50
		Medium	77	7	65
		High	73	11	80
	SSHA	Low	86	2	50
		Medium	82	6	65
		High	78	10	80
	BFGMAQENN_WSBWO	Low	91	1	50
		Medium	88	4	65
		High	84	8	80
**KYOTO**	LA-HLRW	Low	84	2	50
		Medium	80	6	65
		High	75	11	80
	SSHA	Low	88	1	50
		Medium	85	4	65
		High	81	8	80
	BFGMAQENN_WSBWO	Low	97	0.5	50
		Medium	94	3.5	65
		High	90	7.5	80

**Fig. 9:**
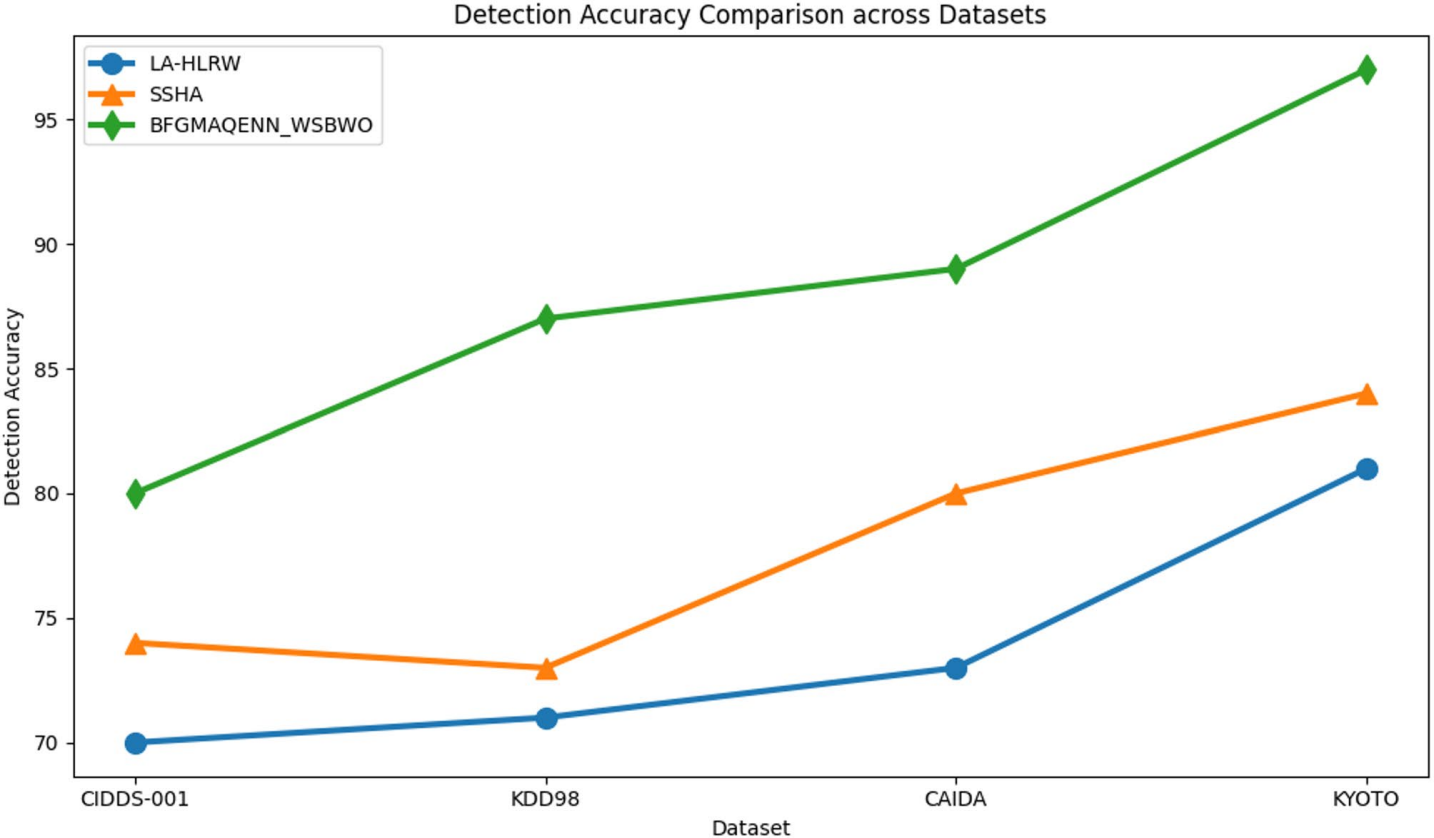
Detection of accuracy by varying the convergence of various datasets.

**Fig. 10:**
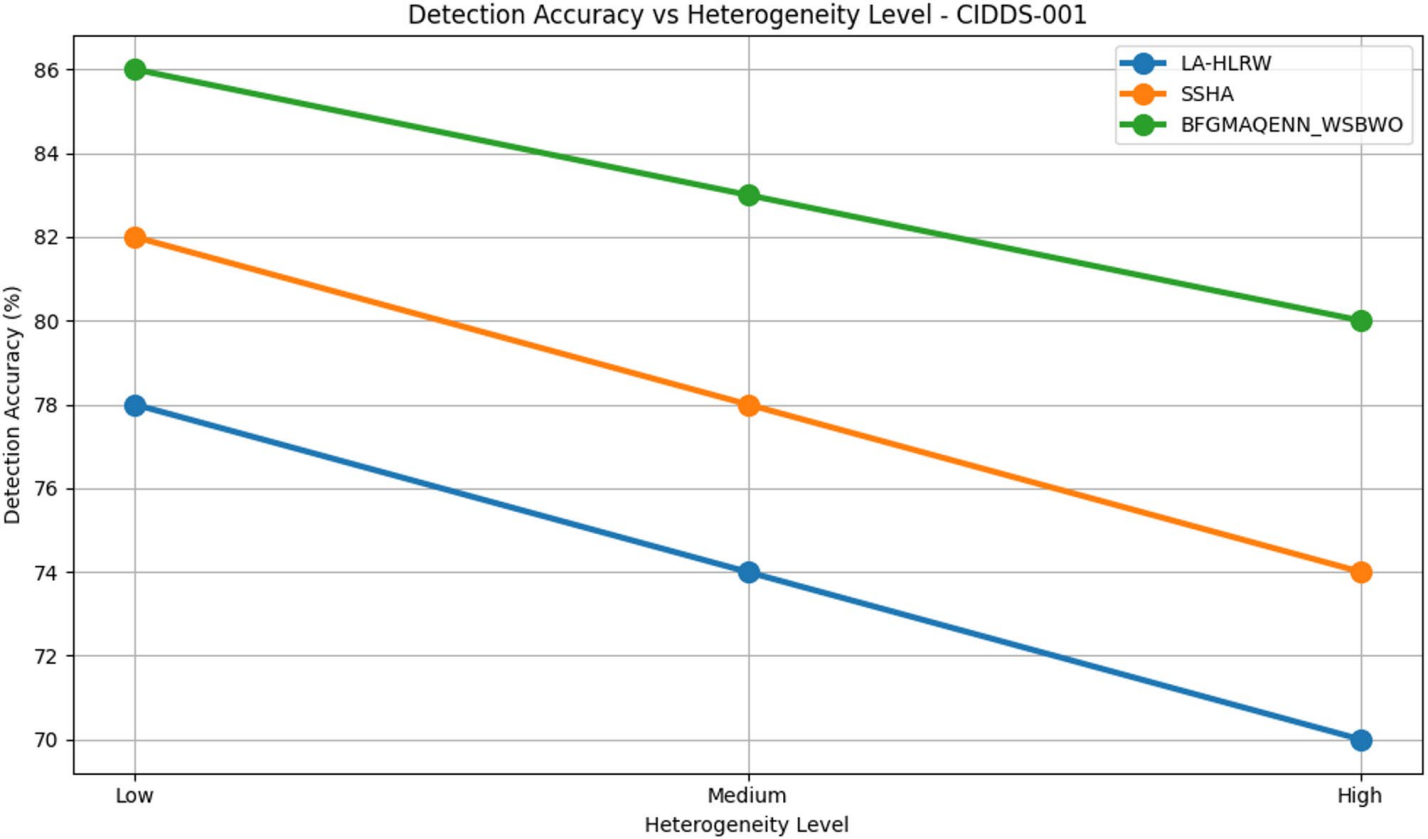
Detection of accuracy vs heterogeneity level analysis on CIDDS-001 Dataset.

**Fig. 11:**
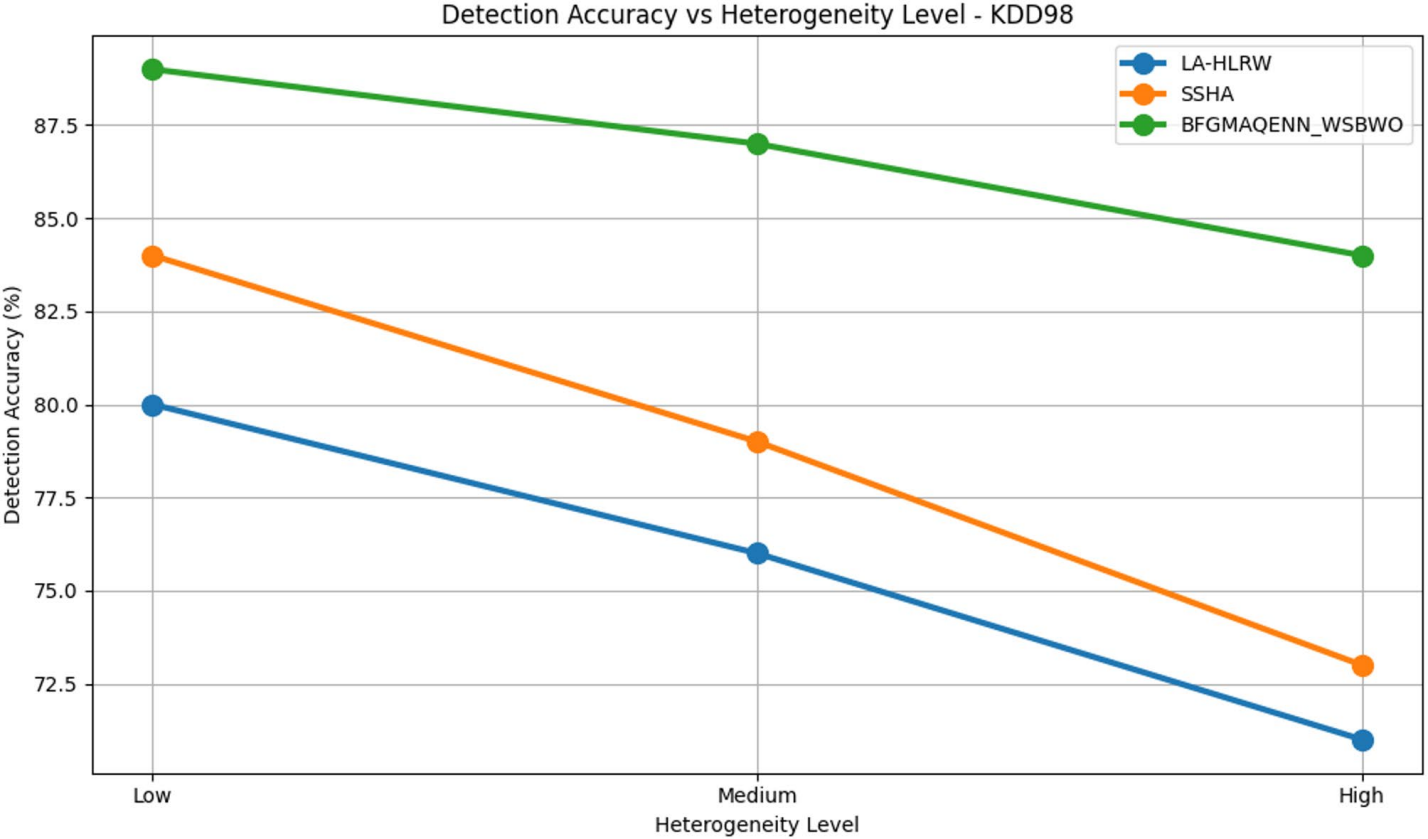
Detection of accuracy vs heterogeneity level analysis on KDD98 dataset.

**Fig. 12:**
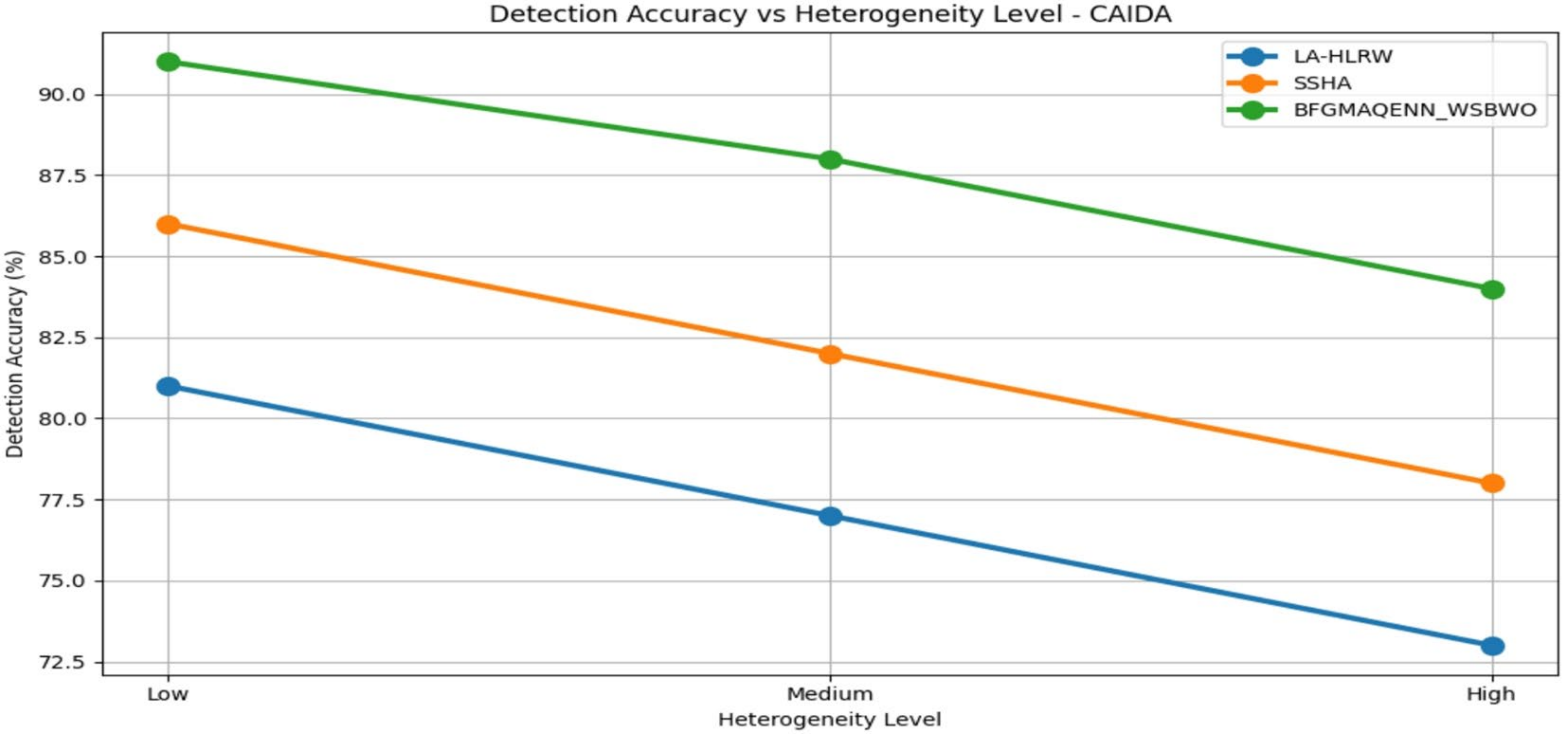
Detection of accuracy vs heterogeneity level analysis on CAIDA dataset.

**Fig. 13:**
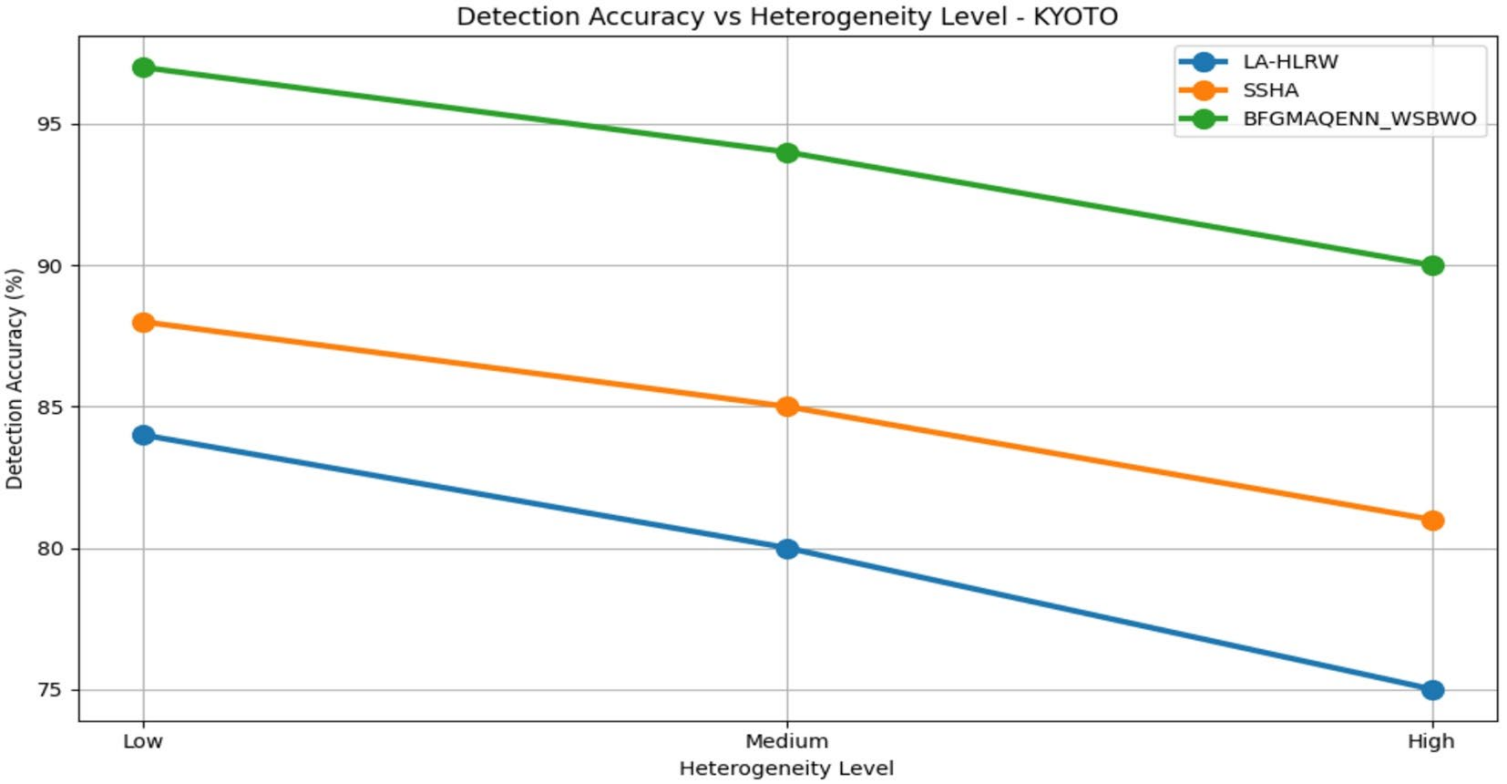
Detection of Accuracy Vs Heterogeneity Level Analysis On KYOTO Dataset.

Table 5 compares the performance of three techniques—LA-HLRW, SSHA, and BFGMAQENN_WSBWO—across four benchmark datasets (CIDDS-001, KDD98, CAIDA, and KYOTO) under varying heterogeneity levels (Low, Medium, and High). The performance is assessed using three metrics: Detection Accuracy (%), Accuracy Degradation (%), and Convergence Lag (Rounds). Across all datasets, BFGMAQENN_WSBWO consistently achieves the highest detection accuracy and the lowest accuracy degradation, regardless of heterogeneity. For example, in the KYOTO dataset, it attains 97% accuracy at low heterogeneity with just 0.5% degradation and maintains a high accuracy of 90% even at high heterogeneity. Convergence lag remains stable at 50, 65, and 80 rounds for low, medium, and high heterogeneity, respectively, across all techniques as shown in Fig. 9. SSHA demonstrates moderate performance, outperforming LA-HLRW but falling short of BFGMAQENN_WSBWO. Its detection accuracy typically ranges in the mid-80s for low heterogeneity and decreases with higher heterogeneity. For instance, in the CAIDA dataset, SSHA achieves 86% accuracy at low heterogeneity but drops to 78% at high heterogeneity, with degradation increasing from 2% to 10%. LA-HLRW consistently records the lowest detection accuracy and higher degradation rates compared to the other two methods. Accuracy reductions are most pronounced under high heterogeneity. In the KDD98 dataset, accuracy declines from 80% at low heterogeneity to 71% at high heterogeneity, while degradation increases from 2% to 11%. Figures 10, 11, 12 and 13 shows that a common trend across all datasets and techniques is that increasing heterogeneity results in reduced detection accuracy and increased accuracy degradation. However, the degree of impact varies by technique: BFGMAQENN_WSBWO shows the most resilience, SSHA displays moderate robustness, and LA-HLRW is most affected.

### Decision-making for security classification

Table 6, shown that the interpretability analysis using SHAP and LIME highlights the most influential features contributing to the detection of malicious traffic across multiple datasets. In the CIDDS-001 dataset, attributes such as *Bytes* and *Flags* were critical in identifying brute force attacks, while unusual *Src Ports* further pointed to scanning activities. For the KDD98 dataset, inconsistencies in *Protocol* and *Service* combined with abnormal *Duration* values were key indicators of intrusion attempts.

**Table 6:** Highlights top five contributing features across datasets via SHAP and LIME.

Dataset	Top Features (via SHAP and LIME)	Interpretability Insight
CIDDS-001	Bytes, Flags, Src Port, Dest IP, Duration	High Bytes with unusual Flags indicated brute force; specific Src Ports suggested malicious scans.
KDD98	Protocol, Duration, Service, Src Bytes, Count	Service mismatches and abnormal Duration strongly linked to intrusions.
CAIDA	Packets, Bytes, Src IP, Dest Port, Flags	Sudden spike in Packets/Bytes linked to DDoS patterns.
KYOTO	Duration, Src Port, ToS, Proto, Bytes	Extended Duration and uncommon Src Ports were strong predictors of malicious activity.

The CAIDA dataset revealed that sharp increases in *Packets* and *Bytes*, along with suspicious *Src IPs* and *Dest Ports*, were closely associated with DDoS activity. In the KYOTO dataset, long *Duration* sessions and uncommon *Src Ports* emerged as strong markers of stealthy denial-of-service and scanning behaviors. Collectively, these results show that specific traffic attributes consistently provide valuable signals for identifying attacks, thereby improving the interpretability, trust, and practical relevance of intrusion detection models in 6G network environments.

**Table 7:** SHAP and LIME percentage contributions for malicious node detection.

Dataset	Top Feature 1 (SHAP and LIME %)	Top Feature 2 (SHAP and LIME %)	Top Feature 3 (SHAP and LIME %)	Top Feature 4 (SHAP and LIME %)	Top Feature 5 (SHAP and LIME %)	Malicious Node Likelihood (%)
**CIDDS-001**	Bytes (**28%**)	Flags (**22%**)	Src Port (**18%**)	Dest IP (**17%**)	Duration (**15%**)	**82%** (Brute Force/Malware)
**KDD98**	Protocol (**25%**)	Duration (**23%**)	Service (**20%**)	Src Bytes (**17%**)	Count (**15%**)	**85%** (Intrusion Detected)
**CAIDA**	Packets (**30%**)	Bytes (**25%**)	Src IP (**20%**)	Dest Port (**15%**)	Flags (**10%**)	**88%** (DDoS Attempt)
**KYOTO**	Duration (**27%**)	Src Port (**25%**)	ToS (**18%**)	Proto (**16%**)	Bytes (**14%**)	**90%** (Stealth DoS/Scan)

**Fig. 14:**
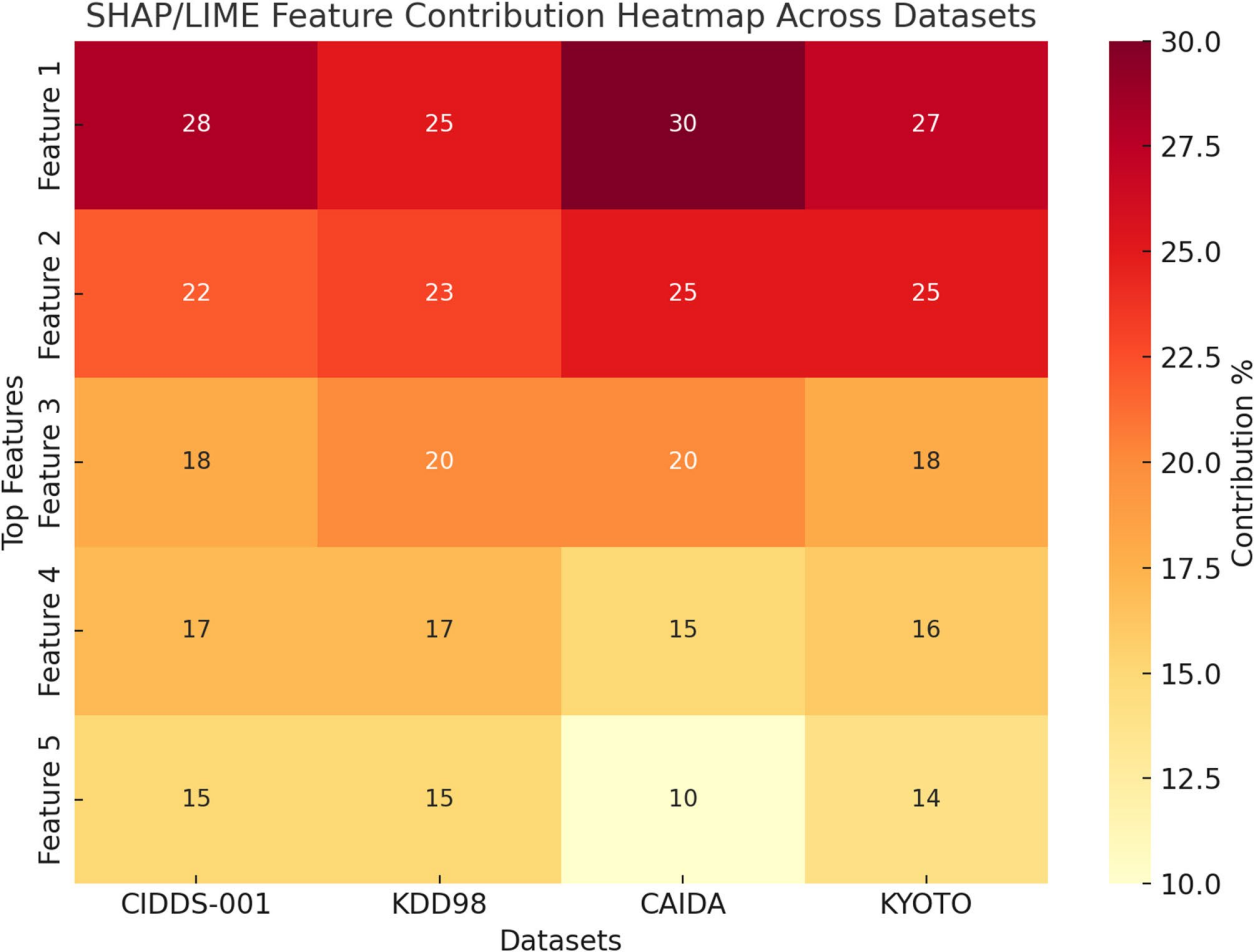
Interpretability analysis of proposed BFGMAQENN-WSBWO Model Using SHAP/LIME.

Table 7 lists the top five contributing features for each benchmark dataset (CIDDS-001, KDD98, CAIDA, and KYOTO) together with their relative influence and the likelihood of a node being flagged as malicious. The analysis shows that Bytes and Flags are dominant indicators in CIDDS-001, Protocol and Duration carry higher weight in KDD98, Packets and Bytes are decisive in CAIDA, while Duration and Src Port are most critical in KYOTO. Figure 14 provides a heatmap visualization of these feature contributions, making it easier to observe dataset-specific variations. Overall, the findings suggest that irregular traffic characteristics—such as abnormal spikes in packet/byte counts, protocol inconsistencies, and uncommon port or duration values—play a key role in identifying malicious behavior. This interpretability layer strengthens transparency, trust, and accountability of intrusion detection in 6G environments.

### Security attack analysis

**Table 8:** Security attack analysis against the proposed method.

Attack Type	Dataset	Accuracy (No Defense)	Accuracy (With Proposed Technique Defense)
Backdoor	CIDDS-001	70	79.4
	KDD98	74	81.3
	CAIDA	80	86.4
	KYOTO	71	87.6
Data Poisoning	CIDDS-001	73	85.4
	KDD98	87	88.2
	CAIDA	73	86.1
	KYOTO	80	84.9
Model Evasion	CIDDS-001	89	89.4
	KDD98	81	87.6
	CAIDA	84	88.9
	KYOTO	97	98.2

Table 8 presents the impact of the proposed defense technique on classification accuracy across four datasets—CIDDS-001, KDD98, CAIDA, and KYOTO—under three different attack types: Backdoor, Data Poisoning, and Model Evasion. The results are compared between scenarios without defense and with the proposed defense in place.

**Fig. 15:**
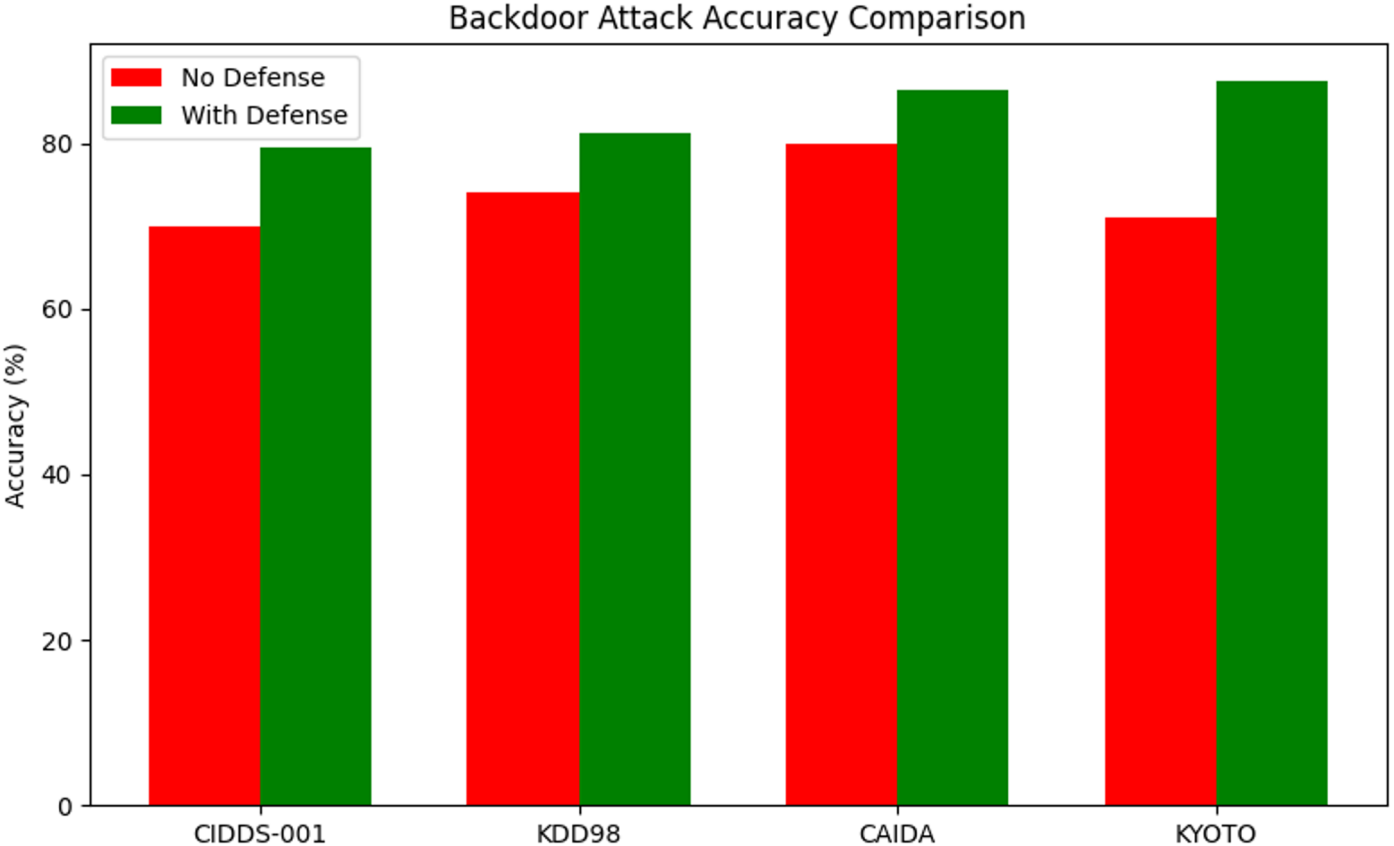
Backdoor attack accuracy comparison against various datasets.

Figure 15 illustrate that, the proposed defense yields substantial accuracy improvements across all datasets. In CIDDS-001, accuracy rises from 70% to 79.4%, while KDD98 improves from 74% to 81.3%. CAIDA shows a similar gain, moving from 80% to 86.4%. Notably, KYOTO exhibits the largest improvement, jumping from 71% to 87.6%, indicating strong resilience against backdoor threats.

**Fig. 16:**
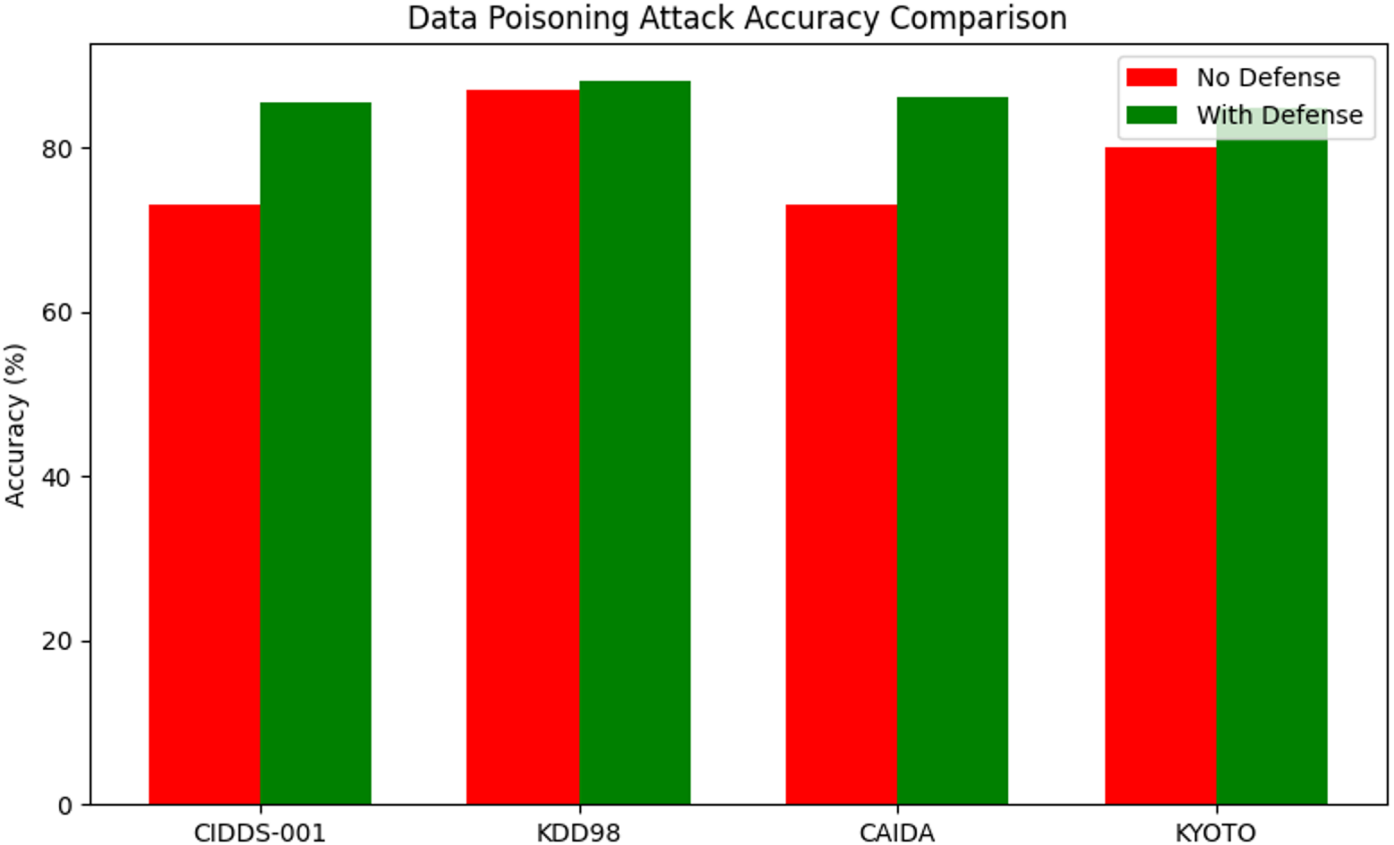
Data poisoning accuracy comparison against various datasets.

Figure 16 illustrate that; the defense is particularly effective in datasets with lower baseline accuracy. For CIDDS-001, performance increases significantly from 73% to 85.4%. KDD98, which already had a high baseline of 87%, shows a modest improvement to 88.2%. CAIDA gains from 73% to 86.1%, while KYOTO sees a rise from 80% to 84.9%. These results highlight the method’s effectiveness in mitigating training data manipulation.

**Fig. 17:**
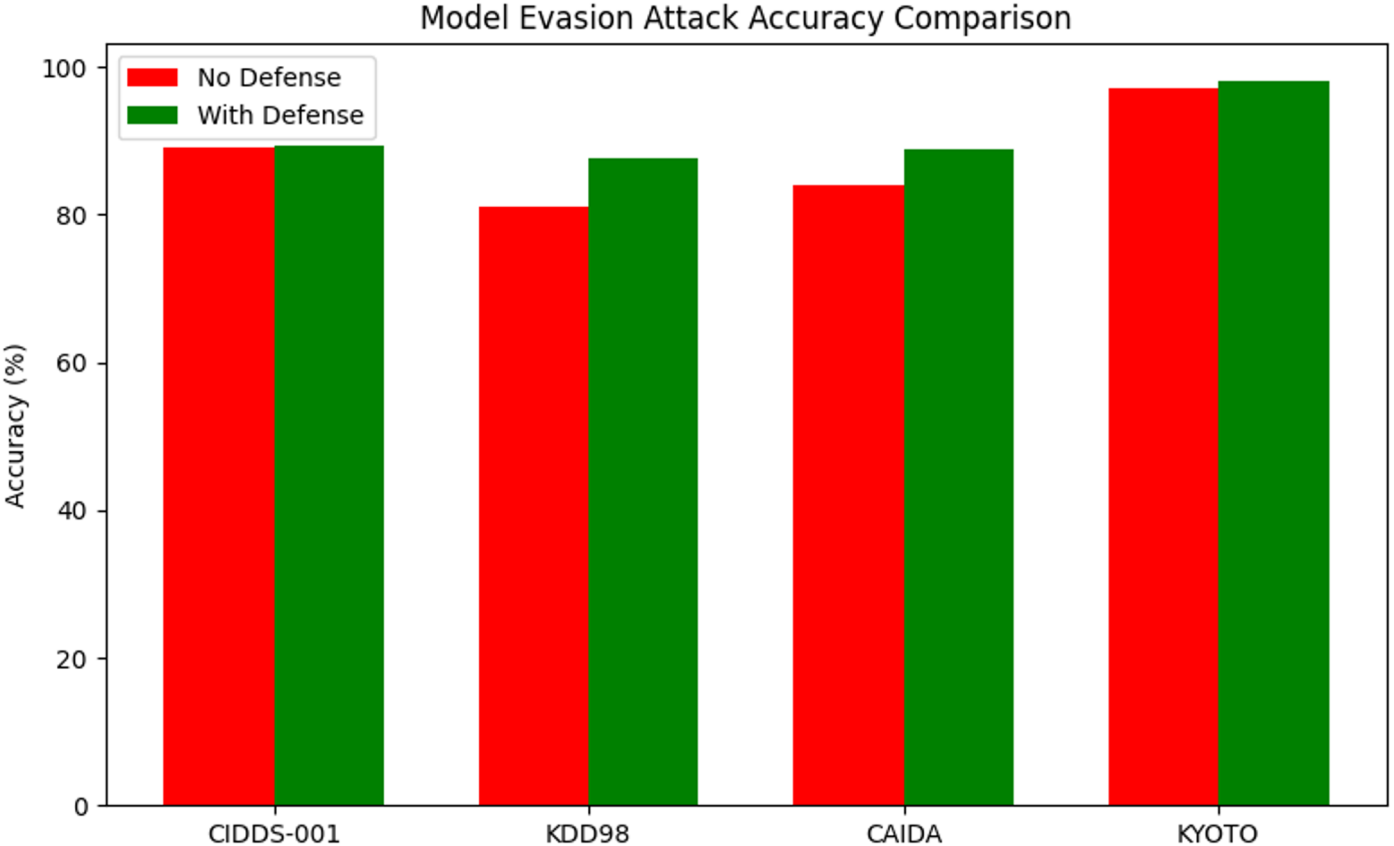
Model evasion attack accuracy comparison against various datasets.

Figure 17 illustrate that, the model evasion attacks on CIDDS-001 shows a slight increase from 89% to 89.4%, while KDD98 experiences a notable rise from 81% to 87.6%. CAIDA moves from 84% to 88.9%, and KYOTO achieves near-perfect accuracy, improving from 97% to 98.2%.

### Communication overhead analysis

**Table 9:** Communication overhead Analysis.

Model update frequency (rounds)	Detection accuracy (%)	Detection latency (ms)	Communication overhead reduction (%)
5	97	15	40
10	96.8	20	50
20	96.3	30	60
40	95.5	50	72
80	94.2	85	85

**Fig. 18:**
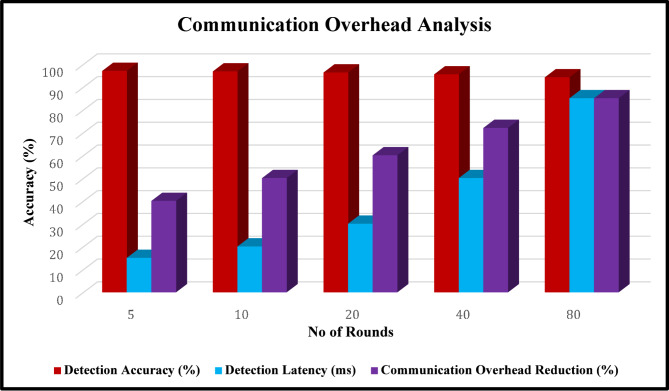
Communication Overhead Analysis.

Table 9, highlights the relationship between model update frequency, detection accuracy, latency, and communication overhead reduction. Figure 18 shows that, when updates occur frequently, such as every 5 rounds, the system achieves the highest accuracy of 97% with minimal latency (15 ms), but communication savings are limited to 40%. Increasing the interval to 10 or 20 rounds slightly lowers accuracy to 96.8% and 96.3%, respectively, while improving communication efficiency to 50–60% and moderately increasing latency. At 40 rounds, accuracy declines further to 95.5%, latency rises to 50 ms, and communication savings reach 72%. The longest interval, 80 rounds, provides the highest communication reduction (85%) but results in the lowest accuracy (94.2%) and the highest latency (85 ms).

### Discussion

This proposed work involves keeping track of the random data packet exchange interface for federated learning connected to the gateway to ascertain whether excessive resource consumption results in the loss of shared weights from each distributed device, thereby compromising the global methodology of federated learning. We confirmed how packet loss during transmission affects the accuracy of attack detection. Packet loss of the suggested model is consistently more than during flooding attacks, even if FL achieved a packet loss of about 90%, as we had anticipated. A blockchain-based system needs every member to re-mine until shared specifications are achieved, even though packet loss occurs during weight transmission. As a result, a higher packet loss does not indicate that weight parameters have not reached gateway.

Furthermore, the suggested model exhibits a low packet loss for every attack, confirming the stability of our suggested approach. Generally speaking, detection accuracy is inversely correlated with packet loss of every attack type. This collaboration improve accuracy of attack detection, guarantees stability of the entire training process, speeds up its pace of convergence. In order to achieve fast convergence and similar accuracy, our AHFL approach helps alleviate the bottleneck caused by data distribution problems in 6G/- massive IoT networks, where every edge server has various training data. It prevents waiting for slow-edge servers, which could result in a lengthy training period.

## Conclusion

This paper presents a novel approach to monitoring cyberattacks in 6G networks and intrusion detection using artificial intelligence technologies. As a means to monitor and provide security to cyberattacks against the 6G system, Blockchain federated Gaussian multi-agent Q-encoder neural networks (BFGMAQENN) have been deployed. Finally, Whale Swarm Binary Wolf Optimization (WSBWO) was applied to enhance the 6G technology. Enhancing the security posture of 6G networks combines collaborative intrusion detection, blockchain for information sharing, and AI-based detection techniques. Representing NextGen IDS in massive IoT networks with 6G support that uses two-phase intelligent attack detection consisting of high-level intrusion detection, lightweight detection, and adaptive mitigation. The next step was to build an HFL architecture for IDSs that could increase scalability and boost communication effectiveness. Compared to traditional FL methods, experiment results demonstrated how well our suggested designs performed in terms of quicker convergence and stable training loss, particularly asynchronous. Proposed model attained detection accuracy of 97%, data integrity of 94%, scalability of 93%, communication overhead of 60%, and network efficiency of 98%. In future research, we will improve the model’s performance by resolving the dataset’s class imbalance. It will need more than just relabelling to provide a balanced dataset. Combining the best features of both systems aims to minimize false positives and detect novel threats or anomalous activity.

## Data Availability

The datasets used and/or analysed during the current study available from the corresponding author on reasonable request.

## Code availability

The relevant code with the manuscript is also available and would be available, if will be asked to do so later.
